# Dosage-sensitive miRNAs trigger modulation of gene expression during genomic imbalance in maize

**DOI:** 10.1038/s41467-022-30704-x

**Published:** 2022-05-31

**Authors:** Xiaowen Shi, Hua Yang, Chen Chen, Jie Hou, Tieming Ji, Jianlin Cheng, James A. Birchler

**Affiliations:** 1 College of Agriculture and Biotechnology, Zhejiang University, Hangzhou, China; 2 Division of Biological Sciences, University of Missouri, Columbia, MO USA; 3 Department of Electrical Engineering and Computer Science, University of Missouri, Columbia, MO USA; 4 Department of Statistics, University of Missouri, Columbia, MO USA

**Keywords:** Gene expression, Plant genetics, Agricultural genetics

## Abstract

The genomic imbalance caused by varying the dosage of individual chromosomes or chromosomal segments (aneuploidy) has more detrimental effects than altering the dosage of complete chromosome sets (ploidy). Previous analysis of maize (*Zea mays*) aneuploids revealed global modulation of gene expression both on the varied chromosome (*cis*) and the remainder of the genome (*trans*). However, little is known regarding the role of microRNAs (miRNAs) under genomic imbalance. Here, we report the impact of aneuploidy and polyploidy on the expression of miRNAs. In general, *cis* miRNAs in aneuploids present a predominant gene-dosage effect, whereas *trans* miRNAs trend toward the inverse level, although other types of responses including dosage compensation, increased effect, and decreased effect also occur. By contrast, polyploids show less differential miRNA expression than aneuploids. Significant correlations between expression levels of miRNAs and their targets are identified in aneuploids, indicating the regulatory role of miRNAs on gene expression triggered by genomic imbalance.

## Abstract (web summary)

The role of miRNAs in genome imbalance is unclear. Here, the authors show that *cis* miRNAs in aneuploids present a predominant gene-dosage effect, whereas *trans* miRNAs trend toward the inverse level by examining global miRNA modulation in haploid and diploid aneuploids with the varied dosage of multiple chromosomal segments in maize.

## Introduction

The concept of genome imbalance has been known for nearly a century in that changing the dosage of individual chromosomes or chromosomal segments (aneuploidy) has more phenotypic defects than changing the dosage of the whole set of chromosomes (ploidy)^1–5^. This phenomenon was presumed to be caused by a dosage effect related to varied gene copy numbers^6–11^. Early studies illustrated that altering the dosage of a gene would result in varied expression of the gene product in different ways. For example, the amount of gene product could be a directly proportional reflection of the varied gene dosage, a phenomenon referred to as a gene-dosage effect^6,7,12^. In addition, dosage compensation refers to the scenario that the amount of gene product in aneuploids could be equivalent to that in the control despite changes in gene dosage^8–10^. Furthermore, an inverse correlation between the chromosomal dosage and the amount of gene product located elsewhere in the genome was observed while examining the modulation of gene expression from the unvaried portion of the genome, known as the inverse dosage effect. In other cases, positive correlations were also found but to a lesser degree^8,10,11,13^.

The molecular functions of the genes that are responsible for these dosage effects were identified, including transcription factors (TFs), components of signal transduction, and chromatin proteins, which are typically members of macromolecular complexes and multicomponent DNA-binding complexes^11,14–21^. A gene balance hypothesis (GBH) was proposed based on these findings. It states that changing the stoichiometry of members of multisubunit complexes will affect their mode of assembly, kinetics, and function of the whole, thus resulting in negative fitness consequences^22–25^. If these multisubunit complexes are composed of TFs or components of signal transduction cascades that affect targets (e.g., other TFs), varying a portion of the genome would be predicted to influence the global gene expression^14^. Indeed, such effects have been found in large-scale biology studies in many organisms such as *Drosophila*, *Arabidopsis*, human, and maize (*Zea mays*), and in reanalysis of yeast and mouse data^26–36^. A greater spread of modulation was observed in the aneuploids than in the ploidy series in studies of global gene expression modulations using mRNA-sequencing (mRNA-seq) in *Arabidopsis* and maize^30,32,34,35^, which matches the phenotypic observation of aneuploids versus the whole-genome ploidy series^2^. These findings further support the GBH that when there is a partial rather than a whole-genomic variation, the stoichiometry of certain subunits relative to the others would be altered, which consequently affects the function of the whole complex. Further, in both *Arabidopsis* and maize, gene expression on the varied chromosome ranged from dosage compensation to a gene-dosage effect, whereas that from the remainder of the genome ranged from no effect to an inverse effect with positive modulations found to a lesser degree^30,32,34,35^. Recent studies of a dosage series of the human sex chromosomes demonstrate global gene modulations of a subset of genes, which were predominantly inverse effects^31,33^. Analyses on global gene expression of autosomal and sex chromosome trisomy in *Drosophila* show that most genes on the varied chromosome present dosage compensation, while the remainder of the genome exhibits widespread inverse dosage effects^28,29,36^.

The GBH is further supported by evolutionary genomic studies. Components of macromolecular interactions including TFs and signal transduction genes are preferentially retained as duplicates for a longer period of evolutionary time after whole-genome duplication (WGD)^21,37–46^. The loss of a member of a duplicated pair can be expected to have profound deleterious effects on the fitness of plants and thus would be selected against^22,42^. Interestingly, over 1/3 of ancestral microRNA (miRNA) positions were retained at both homoeologous sites following WGD in grass species including rice, sorghum, maize, and *Brachypodium*^47,48^. The proportion of retained miRNA sites was greater than that for protein-coding genes in maize, or close to that for TFs in rice^47,48^. Grass miRNAs were found to preferentially target genes involved in regulatory and metabolic pathways^47,48^. Further, most retained miRNA families were associated with retained target genes in all four grass species^48^. Considering that most retained genes are TFs and signal transduction genes^21,37–46^, this phenomenon suggests that miRNAs are involved with complex regulatory networks. Thus, mutations in miRNAs were postulated to have a detrimental effect on plant growth and development, consistent with the observation of biased retention of regulatory genes. Therefore, miRNAs in grass species are important regulators in transcription/translation that are dosage-sensitive and possibly function similarly to TFs under genomic imbalance.

miRNAs are a class of 20- to 24-nt endogenous small non-coding RNAs that control gene expression through translational inhibition and mRNA target cleavage^49^. Primary miRNA (pri-miRNA) transcripts are transcribed by RNA polymerase II from the *MIR* gene, subsequently excised from stem-loop structures by Dicer-like 1 (DCL1), 2’-*O*-methylated by Hua-Enhancer 1 (HEN1) to produce mature miRNA, exported to the cytoplasm by the Hasty (HST) protein, and then loaded into the Argonaute (AGO) component of an RNA-induced silencing complex (RISC)^49^. Recent studies demonstrated that miRNAs can be exported to the cytoplasm after assembly into RISC by CRM1/EXPORTIN1 (EXPO1), while HST also regulates pri-miRNA transcription and processing^50,51^. miRNAs play critical regulatory roles in various biological processes in plants, including growth, development, cell fate, and stress responses^52–56^. A great deal of interest has been placed on the dosage effect of miRNAs in mammals due to their importance in human diseases. Changes in global miRNA abundance were found in multiple human cancers^57,58^. Subtle alteration of miRNA dosage had profound consequences for the development and maintenance of the heart in mice^59^. A recent study reported that dysregulation of global miRNA dosage control affected lipid metabolic pathways and interfered with embryonic development by disrupting germ layer specification in mammalian cells^60^. In a study of vertebrates, miRNA genes were retained from the WGDs but not from tandem events illustrating stoichiometric constraints^61^. These results imply the importance of precise dosage control of mammalian miRNAs.

Despite all the advances in the study of the dosage effect of miRNAs in mammals, little is known about the impact of dysregulated miRNA dosage in plants. miRNA pathways in plants and animals might have undergone parallel evolution and are different in miRNA biogenesis and mode of action^62–64^. Therefore, it is possible that the dosage effect of plant miRNAs is different. It is also unclear how the variation of the partial genome or whole-genome doses affects miRNA expression on a global scale and on a per miRNA basis. To address these questions, small RNA sequencing (sRNA-seq) is performed on the maize mature leaf tissue in this work of a collection of 20 maize B–A translocation lines created by translocations between the normal chromosomes and the B chromosome^65–68^ containing various copies of chromosomal segments covering 82.1% of the maize W22 genome, in concert with a whole-genome ploidy series of haploid, diploid, triploid, and tetraploid lines. Further, interactions between miRNAs and their predicted gene targets are examined because small RNAs (sRNAs) in this study are co-extracted with mRNAs in the recent comprehensive studies of global gene modulation in maize aneuploids and polyploids^34,35^. The results provide insight into the impact of genomic imbalance on miRNA expression and the role of miRNAs in the unbalanced regulatory network.

## Results

### Aneuploidy causes changes in miRNA expression both in *cis* and in *trans*

A collection of 20 maize B–A translocation lines containing various copies of chromosomal segments was screened and verified by fluorescence in situ hybridization (FISH) as described in previous studies^34,35^. We analyzed 15 diploid aneuploids with 1–3 copies for 1S (the short arm of chromosome 1), 1L (long arm of chromosome 1), 3S, 3L, 4S, 4L, 5L, 6L, 7L, 8L, 9S, and 9L, 2–4 copies for 6S and 1–4 copies for 5S and 10L (10L18), and 17 haploid aneuploids with 1-2 copies for 1S, 1L, 2S, 2S_deletion (which contains a deleted part compared to 2S), 3S, 3L, 4S, 4L, 5S, 5L, 6L, 7S, 7L, 8L, 9S, 9L, and 10L (10L19). The breakpoint of TB-10L18 is very close to that of TB-10L19, which are only 0.5 Mb apart^34,35^. These lines are called distal aneuploids as their segmental aneuploidy does not include the respective centromeres. In addition, trisomies and tetrasomies with breakpoints spanning the centromere of chromosome 4, named Proximal Duplication 4 (Dp4)^69^ as interstitial segmental aneuploids, produced by overlapping reciprocal B–A translocations, were examined. Further, a collection of polyploids including haploids, triploids, tetraploids, and their corresponding diploid controls were also assayed. The grouping information and read mapping statistics of these plants are listed in Supplementary Data 1. Monosomies, trisomies, tetrasomies, and their corresponding diploid controls in each aneuploid were referred to as 1D, 3D, 4D, and 2D, respectively, whereas disomies and their haploid controls were named h2D and h1D. In addition, haploids, triploids, and tetraploids and their diploid controls in the ploidy series were named 1X, 3X, 4X, and 2X.

45-day-leaf tissue of the above-mentioned materials was used to examine the effect of aneuploidy and polyploidy on miRNA expression through sRNA-seq. In addition, analysis of W22 diploid plants with one B chromosome (1B) relative to plants without any B chromosome (0B) was used as a control to rule out the effect of the B chromosome on miRNA expression, as some aneuploids contain part or the equivalent of a whole B chromosome or more, whereas their diploid or haploid controls do not have any B chromosome. Reads of all samples were treated and mapped to the W22 reference genome by ShortStack as described in “Methods”^70–73^. Reads per million mapped reads (RPM) normalization was performed and differential gene expression analysis was conducted by Empirical Analysis of Digital Gene Expression Data in R (edgeR)^74,75^. The similarity of miRNA expression levels among biological replicates was determined by principal components analysis (PCA) using normalized read counts (Supplementary Figs. 1 and 2). For all genotypes, values of principal components (PC1 and PC2) for each replicate were within two standard deviations (SDs) from the mean, indicating none of the samples should be considered an outlier. The Pearson correlation coefficient (PCC) between normalized expression levels of each pair of biological replicates was calculated, and the results showed that expression levels of all pairs are significantly correlated (*P* value < 0.05). However, the read length distribution of three sRNA libraries after structural RNAs had been removed did not show distinct peaks at 21 and 24 nt, indicating the lower quality of data (Supplementary Fig. 3). Thus, these samples (4Dp22C5, 1La20, and 1La40) were excluded from further study. Lowly expressed miRNAs with a mean of normalized counts less than 0.5 in experimental and control groups are excluded from further study.

miRNAs for each aneuploid were partitioned into ones that are present on the varied chromosome (*cis*) versus ones that locate in the remainder of the genome (*trans*) according to their locations relative to breakpoints in each B–A translocation line documented in previous studies^34,35^. miRNAs that fall in the *cis* regions of each B–A translocation line are listed, with each line containing 1–7 *cis* miRNAs (Supplementary Data 2). The number of *cis* miRNAs is positively correlated with the size of the corresponding *cis* region (Pearson correlation: *P* value = 0.011, *R* = 0.55) (Supplementary Data 3).

**Fig. 1:**
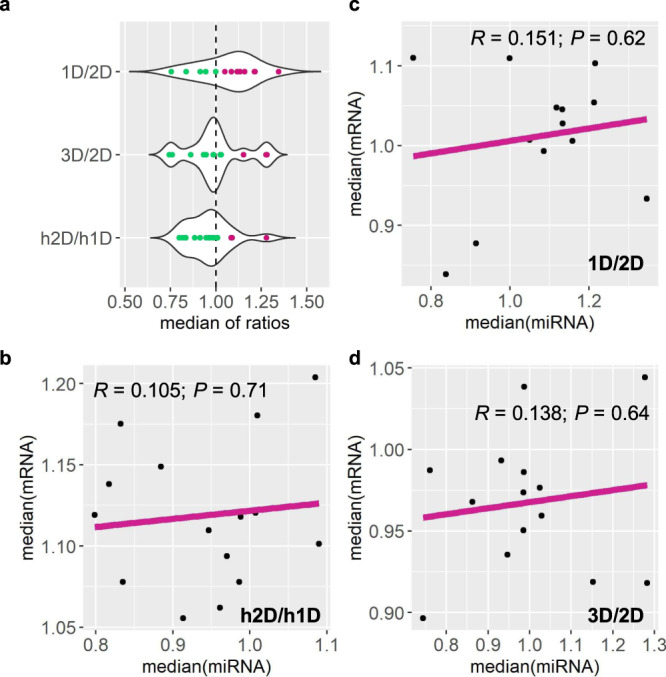
Relationship between expression of *trans* genes and *trans MIRNAs* in each distal aneuploidy comparison. Each data point represents one comparison out of all distal aneuploids assayed. Medians of *trans* miRNA ratios were computed as in Supplementary Data 5. **a** Medians of *trans* miRNA ratios. The x-axis refers to the median of *trans* miRNA ratios, while the y-axis represents the chromosomal dosage in each comparison. Data points that belong to two clusters produced by the *k*-means clustering algorithm were painted in different colors. **b**–**d** Medians of ratios of *trans* miRNAs and *trans* genes in each comparison were plotted on the x- and y-axes, respectively. Medians of *trans* gene ratios were computed in previous studies^34,35^. *R* Pearson correlation coefficient; *P* *P* value for Pearson correlation (two-sided; confidence intervals, 95%; no adjustment made for multiple comparisons). Data points with x- and y-axis values exceeding two SDs from the mean are considered outliers, and thus are excluded from the analysis. Source data are provided as a Source Data file.

Normalized counts of biological replicates were averaged and ratios of individual miRNAs of each experimental condition to the control were computed (Supplementary Data 4). The medians of *trans* ratios of distal aneuploids including monosomies, trisomies, and disomies relative to their corresponding controls were plotted to illustrate the overall trend of *trans* miRNA modulation (Fig. 1a). The medians of ratios distribute over or under a ratio of 1.0, indicating trends of both up-and downregulation exist for *trans* miRNAs triggered by aneuploidy of different chromosomal regions. Further, the finding that the medians of *trans* ratios of miRNAs cluster toward ratios presenting an inverse effect (2.0 for 1D/2D, 0.67 for 3D/2D, and 0.5 for h2D/h1D) suggests that the predominant effect in *trans* miRNAs is an inverse modulation.

**Fig. 2:**
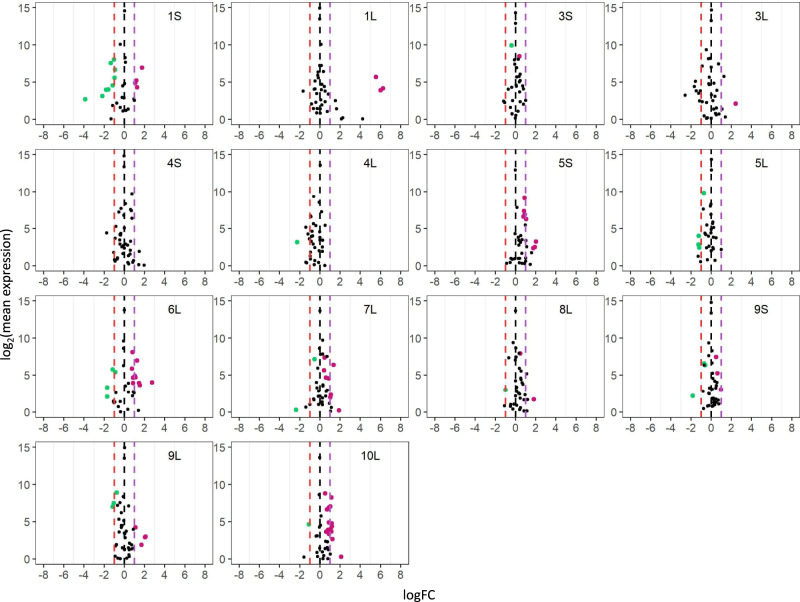
Scatter plots of *trans MIRNA* expression in each monosomy compared with diploids (1D/2D). The x-axis represents the log fold change with base 2 of each experimental genotype to the control, whereas the y-axis notes the log_2_ value of the mean of normalized counts of each experimental genotype and the control. Data points with an FDR < 0.05 and a corresponding logFC > 0 were depicted in magenta, while points with an FDR < 0.05 and a corresponding logFC < 0 were depicted in green. Otherwise, they were painted in black. A fold change (FC) of 1.0 (logFC of 0) represents no change. An FC of 2.0 represents the inverse ratio of *MIRNA* expression in *trans*, whereas 0.5 represents a positive modulation. These FC values are demarcated with labeled vertical lines in red (0.5), black (0), and purple (2.0). Source data are provided in Supplementary Data 4.

**Fig. 3:**
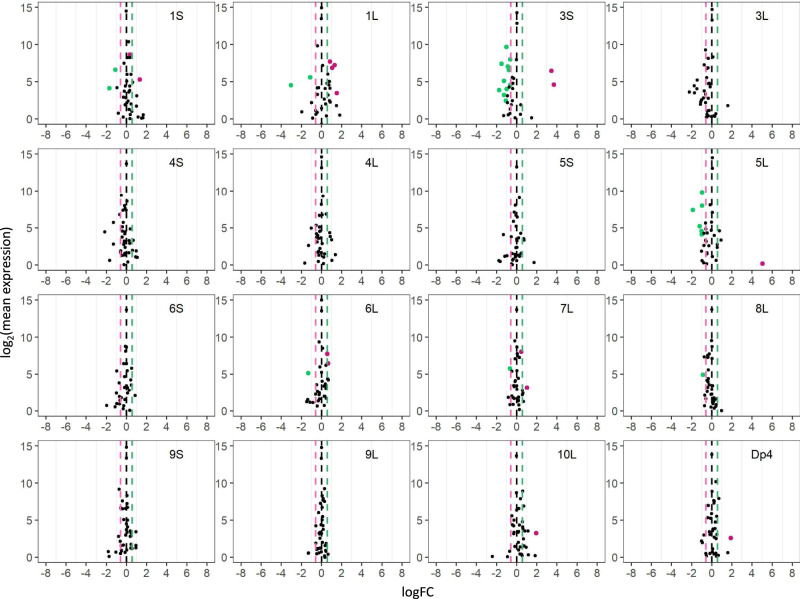
Scatter plots of *trans MIRNA* expression in each trisomy compared with diploids (3D/2D). Analysis was conducted as described in Fig. 2. An FC of 0.67 represents the inverse ratio of *MIRNA* expression in *trans*, whereas 1.5 represents a positive modulation. These FC values are demarcated with labeled vertical lines in pink (0.67), black (0), and green (1.5). Source data are provided in Supplementary Data 4.

**Fig. 4:**
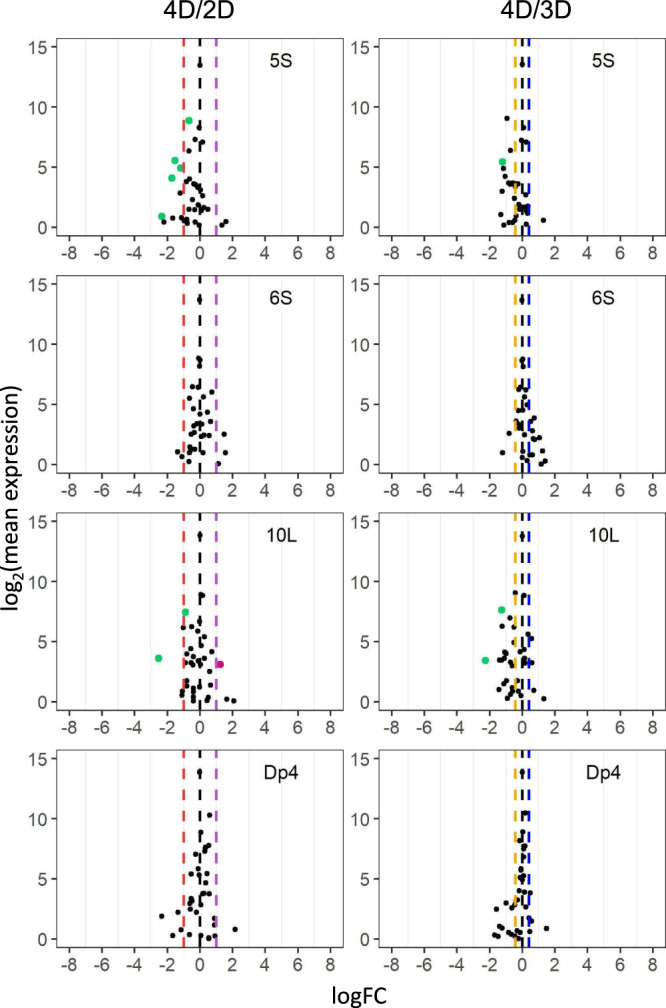
Scatter plots of *trans MIRNA* expression in each tetrasomy compared with diploids (4D/2D) or trisomies (4D/3D). Analysis was conducted as described in Fig. 2. FCs of 0.5 (4D/2D) and 0.75 (4D/3D) represent the inverse ratio of *MIRNA* expression in *trans*, whereas 2.0 (4D/2D) and 1.33 (4D/3D) represent a positive modulation. These FC values are demarcated with labeled vertical lines in red (0.5), orange (0.75), black (0), blue (1.33), and purple (2.0). Source data are provided in Supplementary Data 4.

**Fig. 5:**
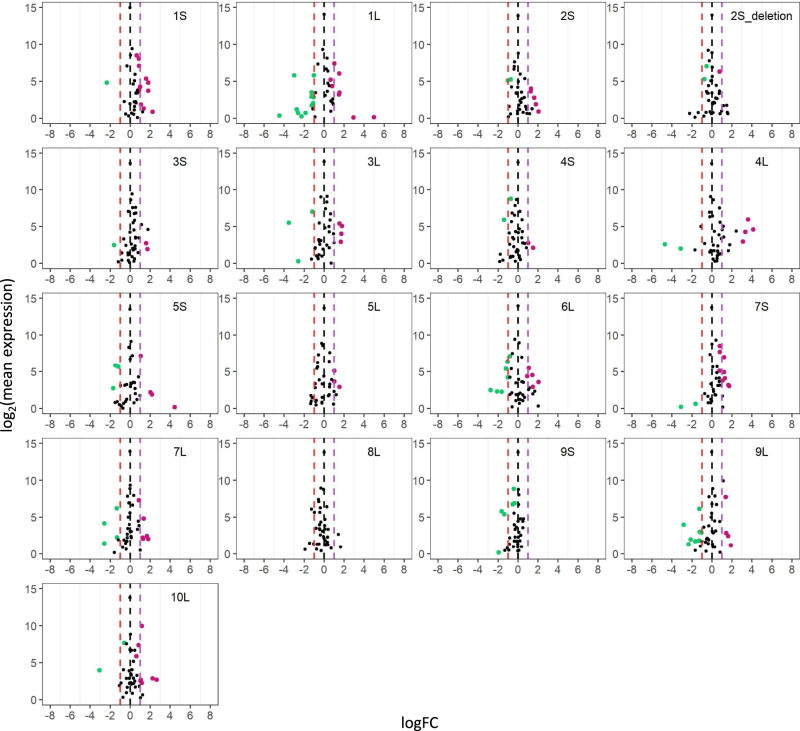
Scatter plots of *trans MIRNA* expression in each disomy compared with haploids (h2D/h1D). Analysis was conducted as described in Fig. 2. An FC of 0.5 represents the inverse ratio of *MIRNA* expression in *trans*, whereas 2.0 represents a positive modulation. These FC values are demarcated with labeled vertical lines in red (0.5), black (0), and purple (2.0). Source data are provided in Supplementary Data 4.

**Fig. 6:**
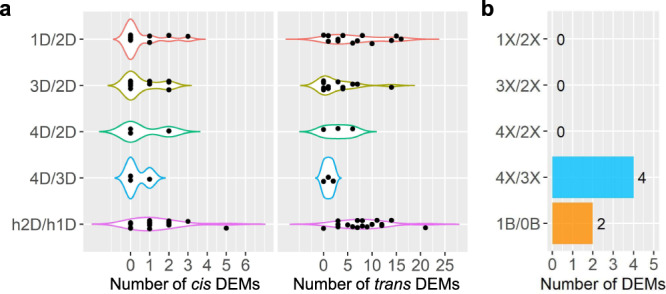
The number of DEMs in each comparison. DEMs were computed by significance (FDR or adjusted *P* value < 0.05), as in each scatter plot in Figs. 2–5, 7. **a** Number of *cis* (left panel) and *trans* (right panel) DEMs in each distal aneuploid compared with the control. Each data point represents the number of DEMs (x-axis) in each comparison (y-axis) as shown in Supplementary Data 3. **b** Number of DEMs in the ploidy series and B chromosome series compared with the control. Source data are provided in Supplementary Data 3.

Scatter plots were used to illustrate the fold change and significance of differential miRNA expression (DME)^30,34,35,76^. Results of the DME analysis of distal aneuploids show differential expression of miRNAs both on the *cis* and *trans* chromosomes compared with their diploid or haploid controls (Figs. 2–5 and Supplementary Data 4). Numbers of differentially expressed *cis* and *trans* miRNAs for each comparison (FDR < 0.05) were shown in Fig. 6a and Supplementary Data 3, with the disomies (h2D/h1D) and monosomies (1D/2D) bearing a greater number of differentially expressed miRNAs (DEMs) compared to aneuploids in other comparisons. There is a significant positive correlation between the number of *trans* DEMs in each B–A translocation line and the corresponding size of the *cis* region for disomies relative to haploid controls (h2D/h1D) (Pearson correlation, *R* = 0.528; *P* value = 0.029), whereas no correlation is observed for comparisons involving diploid aneuploids (Supplementary Fig. 4). Overall, more *cis* DEMs are upregulated in trisomies and tetrasomies relative to diploids, and disomies relative to haploids, whereas more *cis* DEMs are downregulated in monosomies relative to diploids (Supplementary Data 3). The result indicates most *cis* DEMs show a direct dosage effect under the condition of distal aneuploidy.

Monosomies, trisomies, tetrasomies, and disomies act differently in *trans* to some degree in response to changed chromosomal dosage. Scatter plots of DME in comparing monosomies to diploids (1D/2D) show a greater fraction of upregulated *trans* DEMs in most comparisons including 1L, 3S, 3L, 5S, 6L, 7L, 8L, 9L, and 10L, whereas more downregulated *trans* DEMs are observed in 1S, 4L and 5L monosomies (Fig. 2 and Supplementary Data 3). Those in the trisomy/diploid (3D/2D) and tetrasomy/diploid (4D/2D) comparisons display a lesser degree of differential expression, with more downregulated *trans* DEMs for 3S, 5L, and 8L trisomies, and 5S and 10L tetrasomies, whereas there is a greater number of upregulated *trans* DEMs in 1L, 6L, and 10L trisomies (Figs. 3, 4; Supplementary Data 3). The finding of a greater number of *trans* DEMs being modulated towards the direction of an inverse effect for diploid aneuploids further supports the conclusion regarding the predominant inverse effect on *trans* miRNAs. However, DME analysis of disomies exhibits a different trend of *trans* miRNA modulation compared to those of diploid aneuploids. By comparing disomies and haploid controls, 1S, 2S, 3S, 3L, 4L, 5L, 6L, 7S, 7L, and 10L disomies have more upregulated *trans* DEMs, while 1L, 2S_deletion, 4S, 9S, and 9L disomies contain more downregulated *trans* DEMs (Fig. 5 and Supplementary Data 3). By contrast, medians of *trans* ratios for a greater proportion of disomies relative to the haploid control are distributed below the ratio of 1.0 (Fig. 1a and Supplementary Data 5). Medians of *trans* ratios of these disomies are clustered toward the inverse level as determined by the *k*-means clustering algorithm (*k* = 2). In addition, medians of *trans* ratios of many disomies with a greater fraction of upregulated *trans* DEMs are below 1.0 (Supplementary Data 3 and 5). Thus, although slightly more *trans* DEMs are upregulated than being downregulated in disomies relative to haploids, it is still likely that the predominant effect of *trans* miRNA modulation is an inverse effect when analyzed collectively.

There are progressive *cis* and *trans* effects on gene expression in aneuploids, with greater chromosomal dosage producing greater effects as reported in previous studies^34,35^. To examine if such effects are also present for the expression of miRNAs, scatter plots of DME in comparing tetrasomies to trisomies were generated. Although the expression of many miRNAs in the 4D/3D comparison remains unchanged, a few *cis* and *trans* DEMs were identified (Fig. 6a and Supplementary Data 3), indicating the existence of progressive *cis* and *trans* effects on a subset of miRNAs with ascending chromosomal dosage.

Although the predominant effect on global mRNA and miRNA expression in *trans* is an inverse modulation, the medians of *trans* miRNA ratios in this study do not show much correlation with the medians of *trans* mRNA ratios from the corresponding comparisons in previous studies^34,35^, as the Pearson correlation between medians of *trans* ratios of miRNAs and protein-coding genes is not statistically significant as shown in Fig. 1 (*P* value > 0.05). Results from differential mRNA/miRNA expression analysis present a greater proportion of *trans* differentially expressed protein-coding genes (DEGs)/DEMs out of expressed *trans* mRNAs/miRNAs in most conditions (Supplementary Table 1). Thus, the extents of modulation in *trans* miRNAs are different from those of *trans* mRNAs under the influence of varied chromosomal dosages.

**Fig. 7:**
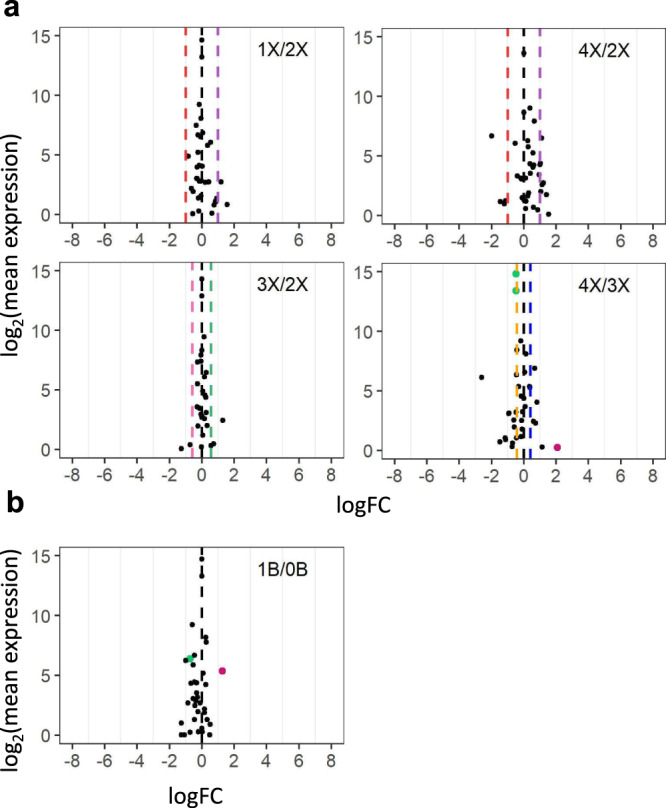
Scatter plots of *MIRNA* expression in each ploidy series and B chromosome series. **a** Scatter plots in each ploidy series compared with diploids. Analysis was conducted as described in Fig. 2. A FC of 1.0 (logFC of 0) represents no change. FCs of 0.5 (1X/2X), 1.33 (4X/3X), 1.5 (3X/2X), and 2.0 (4X/2X) represent a gene-dosage effect, whereas FCs of 2.0 (1X/2X), 0.75 (4X/3X), 0.67 (3X/2X), and 0.5 (4X/2X) represent the inverse ratio of *MIRNA* expression. These FC values are demarcated with labeled vertical lines in red (0.5), pink (0.67), orange (0.75), black (1.0), blue (1.33), green (1.5), and purple (2.0). **b** Scatter plots of diploids with one B chromosome compared with the corresponding control with no B (1B/0B). Source data are provided in Supplementary Data 4.

Apart from the distal aneuploids analyzed above, an interstitial aneuploid line, Dp4, was assayed in terms of different chromosomal dosages. Only 1 DEM was found in comparing trisomies and tetrasomies to their diploid controls (Figs. 3 and 4), indicating varied chromosomal dosage of Dp4 has little impact on *cis* and *trans* miRNA expression. In addition, only 2 DEMs (Cluster_181033 and Cluster_229850) were identified when comparing plants with 1B to those with 0B, implying that the B chromosome is not the major contributor to most miRNA modulation in aneuploids assayed in this study (Figs. 6b and 7b). These 2 miRNAs were excluded from further analysis considering it is difficult to determine whether the impact of aneuploidy on these miRNAs results from aneuploidy of the essential A chromosomal segments or the nonessential B chromosomal segments.

### Polyploids show less differential miRNA expression than aneuploidy

Scatter plots comparing ploidies of haploid, triploid, and tetraploid with the corresponding diploid control show much less modulation compared with aneuploids, with fewer DEMs found in each ploidy comparison than most aneuploidy comparisons (Figs. 6b and 7a). No DEM was identified in comparing ploidies of haploid, triploid, and tetraploid with the corresponding diploid control. It is generally accepted that there is a linear relationship between ploidy, cell size, and transcriptome size in *Arabidopsis* and maize^34,77–79^. Considering that the normalization method used in this study does not reflect the expression level per cell, changes in miRNA expression levels in the ploidy series would be canceled by altered cell size and transcriptome size. Thus, that no DEM was found reflects the minor changes in the relative miRNA expression in the ploidy series.

We further investigated the DME in comparing tetraploids to triploids and found 4 miRNAs to be significantly differentially expressed in the 4X/3X comparison (Figs. 6b and 7a). Analysis of ratios of these 4 DEMs (Cluster_182185, Cluster_211907, Cluster_219450, and Cluster_3815) shows a different trend of modulation in the 3X/2X comparison compared with that in the 4X/2X comparison (Supplementary Data 4). Thus, these DEMs are likely caused by a difference in the genetic background, as triploids originated from inbred line Mo17 while tetraploids were from W22. The results indicate that the trend of miRNA modulation from triploids and tetraploids is not detectable.

### Responses of *cis* miRNAs to changes in chromosomal dosage

**Fig. 8:**
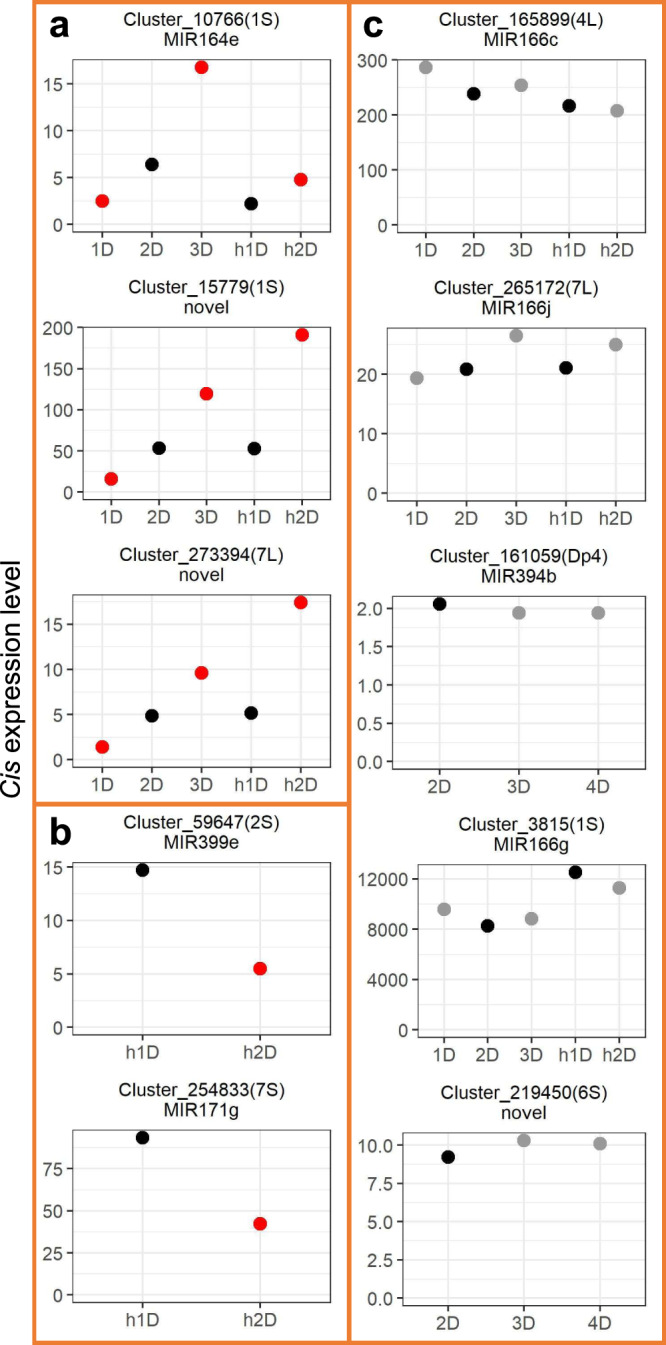
Expression patterns of *cis MIRNAs*. The x-axis refers to the dosage of the *cis* chromosomal region within parentheses, whereas the y-axis designates the *cis* expression level (rpm) of each *MIRNA*. Each *MIRNA* cluster was annotated as described in “Methods”. Novel *MIRNAs* denote *o*nes that failed to match any preexisting annotation. Differential *MIRNA* expression was computed as described in Fig. 2. Control groups were painted in black. *MIRNAs* with FDR < 0.05 compared with the control were depicted in red, whereas ones with FDR ≥ 0.05 were depicted in gray. **a** Example *MIRNAs* showing a dosage effect. **b** Example *MIRNAs* showing a negative dosage effect. **c** Example *MIRNAs* exhibiting dosage compensation. Source data are provided in Supplementary Data 4.

Previous studies examining how diploid aneuploidy influences the expression of individual DEGs revealed four types of responses^34^. Two of them belong to a linear relationship between expression levels and chromosomal dosage, including positive and negative correlations. The other two belong to a nonlinear relationship, in which genes are both upregulated or downregulated in monosomies and trisomies compared to the diploid control, respectively, named increased effect (both upregulated) and decreased effect (both downregulated). It was reported that a gene-dosage effect is the plurality trend for individual *cis* mRNA expression upon changes of chromosome dosage in the range of diploid aneuploid sizes examined^34^. Dosage compensation is also commonly observed for *cis* mRNAs in aneuploids. However, the latter type of response is difficult to be captured considering dosage compensated protein-coding genes are generally not statistically differentially expressed and the insignificance of differential gene expression could be caused by various reasons. To examine if *cis* miRNAs exhibit similar types of responses to chromosomal changes as *cis* mRNAs, the expression pattern of individual *cis* miRNAs was analyzed. Out of the 60 miRNAs that fall in the *cis* region of aneuploids assayed in this study, 45 show a detectable *cis* expression level. Results of DME analysis reveal that 7 *cis* miRNAs are differentially expressed in all aneuploidy conditions available in this study (Fig. 8 and Supplementary Fig. 5, Supplementary Table 2). Five of these miRNAs show a direct gene-dosage effect, while 2 exhibit a negative dosage effect. In addition, 15 are not differentially expressed in any aneuploidy conditions, a few of which show dosage compensation (Fig. 8c). Out of the 23 remaining miRNAs whose expression is differentially expressed in at least one aneuploid condition,11 display a direct gene-dosage effect, as a trend of increased expression with ascending chromosomal dosage was observed. One exhibits a negative dosage effect with a decreased expression upon increased chromosomal dosage. 4 present an increased effect with a greater expression level in aneuploidy compared to diploids and/or haploids, whereas one shows a decreased effect with miRNAs modulated to the opposite direction compared with the increased effect. The 6 remaining miRNAs show a mixed effect with different expression patterns between diploid aneuploidy and haploid aneuploidy (Supplementary Fig. 5). Thus, the direct gene-dosage effect is the predominant type of response of individual *cis* miRNAs reacting to chromosomal changes.

### *Trans*-acting effects of miRNAs under the influence of aneuploidy

**Fig. 9:**
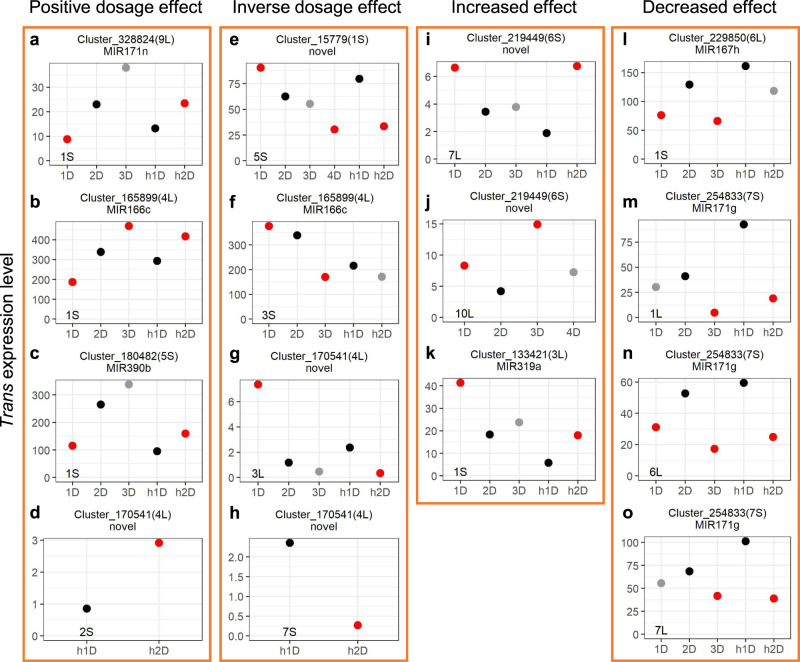
Expression patterns of *trans* DEMs. The *cis* location of each *MIRNA* is depicted within parentheses. The x-axis refers to the dosage of the chromosomal region as noted in the bottom-left corner of each panel, whereas the y-axis designates the expression level (rpm) of each *MIRNA* corresponding to the genotype on the x-axis. Thus, all *MIRNAs* are in *trans*, with altered expression levels and unvaried DNA copy numbers. Other aspects were computed as in Fig. 8. **a**–**d** Example *MIRNAs* showing a positive modulation. **e**–**h** Example *MIRNAs* showing an inverse dosage effect. **i**–**k** Example *MIRNAs* exhibiting an increased effect. **l**–**o** Example *MIRNAs* exhibiting a decreased effect. Source data are provided in Supplementary Data 4.

Predominant increased and decreased effects in which the expression of many DEGs under genome imbalance is modulated toward the same direction regardless of increased or decreased chromosomal dosage were observed for *trans* DEGs in diploid aneuploids, although linear correlations between mRNA expression levels and chromosomal dosage were also detected^34^. By contrast, only the positive dosage effect and inverse dosage effect were observed in *trans* mRNAs in disomies compared with their corresponding haploid controls^35^. To investigate if miRNAs have the same *trans*-acting effects, expression patterns of *trans* DEMs were assayed. *Trans* DEMs that are differentially expressed in disomies compared with haploids when diploid aneuploids are not available, or those that are differentially expressed in all aneuploidy comparisons except one when diploids aneuploids are available (e.g., differentially expressed in 1D/2D and h1D/h2D but not in 3D/2D) were selected for further analysis. 25 *trans* miRNAs with significant differential expression in 1–7 different B–A translocation lines were identified (Fig. 9 and Supplementary Fig. 6). For the 28 *trans* miRNAs differentially expressed in disomies compared with haploids but not available in diploid aneuploids due to differences in chromosome arms analyzed, 21 exhibit a positive dosage effect whereas 7 show an inverse dosage effect (Fig. 9d, h, and Supplementary Fig. 6). Out of the 35 miRNAs differentially expressed in diploid aneuploids (sometimes also in disomies), 8, 6, 3, and 7 *trans* DEMs show a positive dosage effect, inverse dosage effect, increased effect, and decreased effect, respectively, while 11 display a mixed effect that does not belong to any of the four described above (Fig. 9 and Supplementary Fig. 6). Thus, both linear and nonlinear relationships between chromosomal dosage and the direction of genomic modulation exist in the regulation of *trans* miRNAs in aneuploidy.

In addition, *trans* expression of one miRNA expressed in plants with aneuploidy of different *cis* regions shows similar and/or different types of responses. For example, *MIR171g* (Cluster_254833), located in the *cis* region of 7S, displays a decreased *trans* effect in diploid and haploid aneuploidy relative to the control with the varied chromosomal dosage of 1L, 6L, and 7L (Fig. 9m–o). By contrast, *trans* expression of *MIR166c* (Cluster_165899) located on 4L shows a positive dosage effect with the varied dosage of 1S but shows an inverse effect with altered copy number of the chromosomal segment 3S (Fig. 9b, f). Another newly identified miRNA, encoded by Cluster_170541 on 4L, was expressed with a positive effect in 2S aneuploids whereas with an inverse effect in 3L and 7S aneuploids, respectively (Fig. 9d, g, h).

### *Cis* miRNAs function as regulators of gene expression in aneuploidy

To test if miRNAs function as regulators in the changes in global gene expression induced by aneuploidy, correlations between expression levels of miRNAs and their putative targets were examined. Targets of all 64 miRNAs found in this study were predicted by psRNAtarget (Supplementary Data 6), with the two miRNAs impacted by the B chromosome excluded from further study. After data treatment, 768 predicted interactions function through target cleavage between expressed miRNAs and their corresponding targets were identified. The relationship between a miRNA and its target was determined by Pearson correlation between the expression levels of the two across all available genotypes. Expression levels of 192 (25%) of these predicted interactions are significantly correlated as shown in Supplementary Data 7 (Pearson correlation, *P* value < 0.05). As a complement, interactions between miRNAs and their targets were characterized by summarizing data generated from degradome sequencing from previous studies in maize (see Methods). 750 interactions were identified, 167 (22%) of which show a significant correlation across all genotypes (Pearson correlation, *P* value < 0.05; Supplementary Data 8). 49 of these significant interactions could be found by both psRNATarget prediction and degradome sequencing data analysis, 33 of which have a negative PCC value. A Gene Ontology (GO) Enrichment analysis on all targets whose expression levels are identified to be significantly correlated with those of their corresponding miRNAs through psRNATarget prediction or degradome sequencing data analysis revealed an enrichment of terms related to protein refolding (GO:0042026), developmental process (GO:0032502), and regulation of transcription, DNA-templated (GO:0006355) (Supplementary Table 3). Similarly, terms related to transcription, DNA-templated (GO:0006351), developmental process (GO:0032502), and regulation of transcription, DNA-templated (GO:0006355) are enriched in targets from the 49 interactions identified by both psRNATarget prediction and degradome sequencing data analysis (Supplementary Table 4). These results indicate the putative regulatory role of targets modulated by miRNAs under the impact of aneuploidy.

**Fig. 10:**
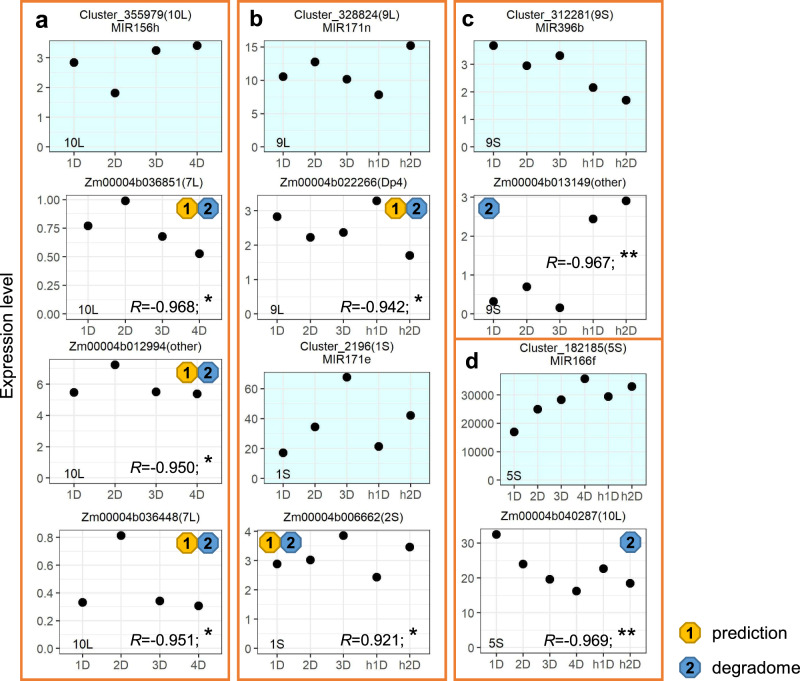
Expression levels of *cis MIRNAs* and their *trans* targets. The *cis* location of each *MIRNA* or each target is depicted within parentheses. “Other” refers to genes that do not fall in the *cis* region of any B–A translocation line. The x-axis refers to the dosage of the chromosomal region as noted in the bottom-left corner of each panel, whereas the y-axis designates the expression level (rpm) of each *MIRNA* in *cis or* each target in *trans* (normalized read counts) corresponding to the genotype on the x-axis. Correlations between *cis MIRNAs* (blue background panel) and their targets (white background panel below each *MIRNA*) were predicted by psRNATarget (yellow circled 1) or gathered from degradome sequencing data from other studies (blue circled 2). *R* Pearson correlation coefficient between expression levels of *MIRNA* and its corresponding target. *P* values for Pearson correlation (two-sided; confidence intervals, 95%; no adjustment made for multiple comparisons): *P value < 0.05; **P value < 0.01 (exact *P* values shown in Supplementary Data 7, 8). **a** Example *MIRNA* that targets SBP domain TFs. **b** Example *MIRNAs* that target GRAS domain TFs. **c** Example *MIRNA* that targets ethylene‐responsive TFs. **d** Example *MIRNA* that targets a mitochondrial-targeted gene Zm00004b040287 (*ZmMORF3*). Source data are provided in Supplementary Data 7, 8.

The results above illustrate that miRNAs function as regulators in global modulations of gene expression in aneuploidy. Now we focus on how *cis* miRNAs impact the expression of *trans* genes on a smaller scale. We assayed the expression levels of the *trans* targets of each miRNA when its dosage is varied in *cis*. PCCs between expression levels of each *cis* miRNA and its predicted *trans* targets in genotypes with the varied dosage of a specific chromosomal segment were computed. miRNAs located in the *cis* region of 2Sa, 2Sa_deletion, 7Sc, and 10L19 are excluded for further analysis because these miRNAs are only expressed in disomies and haploids in *cis* and the corresponding *P* value for Pearson correlation could not be produced. Lowly expressed miRNAs and genes were excluded from the analysis. 27 significant interactions predicted by psRNATarget were identified (Pearson correlation, *P* value < 0.05), 17 of which have a negative PCC value (Supplementary Fig. 7). In addition, 20 significant interactions derived from degradome sequencing data were found (Pearson correlation, *P* value < 0.05), with 13 of them being negative correlations (Supplementary Fig. 8). Five interactions, including 1 positive and 4 negative ones, were characterized by both methods (Fig. 10).

Here we present some examples illustrating how *cis* miRNAs regulate the expression of their *trans* mRNA targets. *MIR156h* (Cluster_355979), located on 10L, shows an increased effect while expressing in *cis*. It negatively regulates the expression of three genes encoding SBP-type domain-containing proteins in *trans* with the varied dosage of chromosomal segment 10L (Fig. 10a). *MIR171n* (Cluster_328824) encodes a miRNA that represses the expression of a gene (Zm00004b022266) encoding a GRAS domain-containing protein in 9L aneuploids. miR171e (Cluster_2196) that shows a direct gene-dosage effect due to changes in the dosage of 1S positively regulates the expression of another GRAS TF (Zm00004b006662) (Fig. 10b). miR396b (Cluster_312281) negatively regulates the expression of a gene (Zm00004b013149) encoding an AP2/ERF domain-containing protein (Fig. 10c). Furthermore, miR166f (Cluster_182185) that exhibits a direct dosage effect in *cis* causes a proportional downregulation of its *trans* mRNA target *MORF3* (Zm00004b040287) that functions in mitochondria (Fig. 10d). These results indicate that varied chromosomal dosages can alter the expression of *cis* miRNAs, thus resulting in changes in the expression of their *trans* mRNA targets.

## Discussion

Despite many recent advances in comprehensive gene expression studies on aneuploidy and polyploidy, the function of miRNAs in genomic imbalance has not been previously investigated. In this study, the global miRNA modulation in haploid and diploid aneuploids with the varied dosage of multiple chromosomal segments was examined in maize, in concert with a whole-genome ploidy series including haploids, diploids, triploids, and tetraploids. Along with the gene expression data collected from previous studies^34,35^, the interactions between miRNAs and their gene targets were assayed. This analysis allows one to address several questions about genomic balance. (1) How do aneuploidy and polyploidy impact the expression of miRNAs both globally and on a per miRNA basis? (2) What is the consequence of varied miRNA dosage in gene expression? (3) Does genomic imbalance similarly affect miRNA expression compared with protein-coding gene expression? This study provides insights into understanding these questions.

Aneuploidy causes varied miRNA expression both in *cis* and in *trans*. While the global trend of *cis* miRNA modulation could hardly be assayed due to the sample-size limit, *trans* miRNAs showed an overall trend of modulation toward the inverse level, although both positive and negative modulations were observed. Such a trend is similar to that of global *trans* protein-coding gene modulation reported in previous studies^34,35^. However, *trans* miRNAs were modulated to a lesser degree in aneuploidy compared with *trans* mRNA genes, indicated by the lack of correlation between medians of ratios of *trans* miRNAs and *trans* mRNAs (Fig. 1) and the proportion of *trans* DEMs out of expressed *trans* miRNAs in contrast to that of DEGs (Supplementary Table 1). Expression of individual *cis* miRNAs shows a predominant dosage effect, although dosage compensation, increased, and decreased effects are also observed. *Cis* genes in previous studies also exhibit similar expression patterns. Analysis of *trans* DEMs reveals the existence of both linear and nonlinear correlations between expression levels of DEMs and the corresponding chromosomal dosage. While the increased and decreased effects are the major trend for *trans* DEGs under genomic imbalance, it is difficult to determine the predominant type of response for *trans* DEMs due to the limitation of the number of miRNAs. Further, for both miRNAs and mRNAs, a lesser degree of modulation was observed in the whole-genome ploidy series than in aneuploidy. In sum, miRNAs are modulated similarly to protein-coding genes under the impact of genomic imbalance.

Apart from the distal aneuploids, the expression of an interstitial aneuploid, Dp4, surrounding the centromeric region of chromosome 4 was examined. Three miRNAs were identified in the *cis* region of Dp4, two of which are expressed in the leaf tissue studied. However, none of them are significantly differentially expressed in Dp4 aneuploids compared with the control. In the previous study, it was found that a larger proportion of *cis* protein-coding genes in Dp4 are dosage compensated than in distal aneuploids, and this phenomenon is not due to epigenetic repression of gene expression around centromeric regions^34^. Considering few miRNAs were identified in the *cis* region of Dp4, it is difficult to determine if the trend toward dosage compensation in *cis* is a result of a specific mechanism or the limit of sample size. Further, while a large number of *trans* DEGs (mRNAs) was found in Dp4 aneuploids, only 1 *trans* DEM (miRNAs) was identified. Ratios of *trans* mRNAs for protein-coding genes in Dp4 aneuploids are distributed toward the inverse level, whereas medians of *trans* ratios of miRNAs are close to 1.0, indicating the trend of regulation of *trans* mRNAs and miRNAs are different in Dp4 aneuploids. These results reveal that varied dosage of Dp4 causes limited changes in miRNA expression both in *cis* and *trans*. Additional heterochromatic regions will need to be examined to determine if the lack of modulation of miRNAs in Dp4 is generalizable for aneuploidy of centromeric heterochromatin.

Regulatory networks that involve both miRNAs and genes in aneuploidy are likely to be complicated. There could be various interactions between a miRNA and its target mRNA as well as the TFs that regulate both. For example, the varied copy number of a miRNA in *cis* could result in altered expression of a TF in *cis* or *trans*, which could lead to altered expression of another protein-coding gene or miRNA in *cis* or *trans*. Considering the lack of information regarding interactions between TFs and their miRNA target in maize and the complexity due to the additive impact on mRNA/miRNA expression from changes in expression of various upstream protein-coding genes/miRNAs, here we restrict our analysis to the simplest interactions. To examine the interaction between miRNAs and their targets in aneuploidy globally, expression levels of miRNAs and their targets were assayed across all genotypes regardless of *cis* and *trans* effects. In addition, to investigate interactions on a smaller scale, the expression of *cis* miRNAs and their *trans* targets with the varied chromosomal dosage of the same region was analyzed. Such type of analysis also provides information regarding whether different types of responses in *trans* protein-coding genes (e.g., inverse effect) are a result of varied expression of miRNAs.

Further, miRNAs control gene expression through RNA target cleavage or translational inhibition^49^. Since RNA-seq experiments do not provide information related to the latter aspect, this study focuses on the regulation of gene expression via RNA target cleavage. In this case, expression of the gene target is postulated to be either correlated with that of the miRNA, or not detectable due to complete gene silencing. However, when genes are totally silenced, their expression levels are approaching zero. Genes not being expressed in RNA-seq data could be caused by various reasons, not necessarily gene silencing. Further, it has been reported that the expression of miRNAs and that of their targets are generally negatively correlated in plants, although positive correlations are also observed^80–82^. In a study identifying miRNA expression-related quantitative trait loci in maize, both positive and negative regulations between miRNAs and their target genes have been characterized^83^. Thus, this study only focuses on interactions when expression levels of miRNAs are statistically significantly correlated with those of their targets predicted by psRNATarget or proven by degradome sequencing.

Associations that involve miRNAs and their targets characterized in this study are also reported in other studies. For instance, the interaction between miR171 and GRAS TFs was identified in *Arabidopsis*, maize, tomato, and *Larix kaempferi*^84–88^. miR171 acts to negatively regulate GRAS TFs in mediating shoot branch production and gibberellin-regulated chlorophyll biosynthesis under light conditions during leaf development in *Arabidopsis*^84,86^. It was reported in many studies that miR156 targets many SBP-box TFs that regulate a large network regulating plant growth and development^89–92^. For example, miR156 targets SBP-box TFs that are required for male fertility in *Arabidopsis*^93^. miR156 was also reported to target SBP-box TFs that interact with other TFs involved in bread wheat tillering and spikelet development^94^.

Expression levels of miRNAs and their targets in 49 interactions identified by both psRNATarget and degradome sequencing present significant correlations across all genotypes, indicating miRNAs function as regulators consistently in aneuploidy with the varied dosage of various chromosomal segments. However, PCCs of these significant interactions fall between −0.53 and 0.70 (Supplementary Data 7, 8). That the strength of the correlations is relatively low is likely caused by the influence of the tangled unbalanced regulatory network on the expression of the targets. When examined on a smaller scale, the varied chromosomal dosage of the same portion of the genome results in different expression patterns of *cis* miRNAs, and their targets in *trans* are thus modulated differently. Effects of *trans* targets include positive correlations, inverse effect, increased, and decreased effects (Fig. 10 and Supplementary Figs. 7, 8). Thus, the four types of responses observed in *trans* DEGs triggered by aneuploidy could be caused by regulation carried out by miRNAs, at least partially. The function of miRNAs in dosage response was also reported in other species. For example, Z-linked miR-2954-3p functions in dosage compensation of the bird Z chromosome, by preferentially down-regulating Z-linked dosage-sensitive genes in males in chicken and zebra finch^95^.

Previous studies on gene expression support the GBH in that genomic stoichiometry plays an important role in quantitative gene expression. This study further reveals the regulatory role of miRNAs fits in the GBH. *Cis* and *trans* miRNAs respond to changes in chromosomal dosage in similar ways to protein-coding genes under the impact of genomic imbalance. In addition, much less modulation of miRNA expression was observed in the whole-ploidy series compared with aneuploids. From the perspective of evolutionary genomics, miRNAs are preferentially retained as duplicates in plants^47,48^. This is likely due to the regulatory role of miRNAs that predominantly target genes involved in regulatory and metabolic pathways, most of which involve multicomponent interactions. Thus, the removal or deletion of a miRNA within a duplicated pair would have negative fitness consequences and would be selected against during evolution. These findings indicate miRNAs are likely to be dosage-sensitive in plants, which is further supported by the results of this study. In addition, the regulatory role of miRNAs in the expression of components of multisubunit complexes has also been verified, as an enrichment of genes involved in transcription was observed for the targets regulated by miRNAs in aneuploidy. Thus, this study reveals miRNAs as participants of the gene regulatory network under genomic imbalance. Varied chromosomal dosage results in modulation of dosage-sensitive miRNAs, and thus causes changes in expression of their targets, which could operate in cascades affecting more genes. Results in this study imply the potential application of changing the dosage of specific miRNAs as regulators of gene expression under genomic imbalance. In other words, modulation of miRNA expression could reduce the genomic imbalance through neutralizing changes in the expression of key dosage-dependent modifiers occurring with aneuploidy. This study also provides a valuable resource for the identification of potential targets of specific miRNAs. Transgenic expression of these miRNAs in maize would facilitate research on the function and molecular mechanism of specific miRNAs.

It has been reported in several studies that aneuploidy of different chromosomal regions results in various phenotypic defects, impacting the floral development and timing, the development of leaf, culm, root, and so on^35,96,97^. Transcriptomic analyses of mRNA-seq and sRNA-seq data in the present and previous studies as well as the phenotypic descriptions of aneuploids are valuable resources for the identification of potential quantitative trait loci^34,35,96,97^. However, relating a phenotypic effect to a specific miRNA, gene, or locus is not easily achieved. Varied copy number of a chromosomal region causes differential expression of hundreds to thousands of genes, likely due to the influence of the tangled unbalanced regulatory network on gene expression^30,34,35^. Considering the coexistence of linear and nonlinear relationships between varied chromosomal dosage and the corresponding gene expression levels^34^, relating changes in miRNA or gene expression to phenotypic effects would require high-quality miRNA and/or gene regulatory networks, or computational tools specifically designed for unraveling regulatory networks in aneuploidy, which still await development in maize. In addition, future studies that dissect the chromosomal regions into smaller blocks would be helpful to reduce the complexity mentioned above.

## Methods

### Plant material, nucleic acid isolation, and sRNA-seq library construction

45-day-old leaf tissue of monosomies, trisomies, and tetrasomies was from a former study with diploids as the control;^34^ whereas that of disomies and their haploid control were from another study^35^. Total RNA and sRNA were co-extracted from maize leaves using mirVana™ miRNA Isolation Kit (Thermo Fisher Scientific, AM1560). Total RNA was used for mRNA-seq in two former studies^34,35^. sRNA libraries were generated directly from total RNA with TruSeq Small RNA Library Preparation Kit (Illumina, RS-200-0048) and were sequenced on the NextSeq500 platform at the University of Missouri Genomics Technology Core. sRNA-seq data of W22 maize plants with one B chromosome and W22 wild-type plants with no B chromosome were from a previous study^98^. The grouping information of the above materials was summarized in Supplementary Data 1.

### sRNA-seq data process

Adaptors at the end of the reads were trimmed using cutadapt version 1.16^99^ and low-quality reads were removed using the FASTX-Toolkit (http://hannonlab.cshl.edu/fastx_toolkit/, fastq_quality_filter -Q33 -q20 -p80). Reads mapped to known structural RNAs (rRNAs, tRNAs, sn-RNAs, and sno-RNAs) from Rfam 14.0^100^ using bowtie 2 were removed^101^. Subsequently, reads of 18-30 nt without poly(A) tails were aligned to the maize reference genome W22v2, the maize chloroplast, and mitochondrial genomes^73,102,103^ using ShortStack version 3.8.3 (-mincov 20)^70–72^. Although the A chromosomal segments with varied dosages may contain various miRNA contents due to their different background, it has been shown that genes encoding miRNAs in maize are conserved across different inbreds^47,83^. In addition, considering all the samples were processed simultaneously for pairwise comparison, only the miRNAs that were found among all the samples at a specific location were identified. *MIRNA* loci were annotated by aligning the sequence of each cluster against known miRNA sequences in miRBase 22^104^ and transcript sequences in the RefSeq database (restricted to *Zea mays*) at NCBI with BLASTN^105^, and by referring to the location of genes annotated in the W22v2 genome^73^ (Supplementary Data 2). RPM normalization was performed and lowly expressed miRNAs (the mean of RPM across all biological replicates from the experimental and control group in each comparison <0.5) were excluded from further analysis. DME analyses were performed using edgeR to test for the significance of fold change between each treatment group to the control group using raw read counts as the input^74,75,106^. Considering triploids originated from inbred line Mo17 while tetraploids were from W22, DME analysis in comparing tetraploids to triploids was performed by a two-sided Student’s *t* test, which tests for the significance of whether the mean log_2_ ratio of 4X/2X differs from the mean log_2_ ratio of 3X/2X. *P* values were adjusted by the Benjamini–Hochberg algorithm for computing false discovery rates. miRNAs with an FDR or adjusted *P* value <0.05 were defined as DEMs. PCA plots were generated in R using normalized read counts.

### Generation of scatter plots

To generate scatter plots, FDR and log fold change with base 2 (logFC) produced by edgeR were used^74,75,106^. The logFC between treatment and control was plotted on the x-axis, while the log_2_ value of the mean of RPM normalized counts of the treatment and control group was plotted on the y-axis. Data points with an FDR (or adjusted *P* value in 4X/3X) < 0.05 and a corresponding logFC > 0 were depicted in magenta, while points with an FDR (or adjusted *P* value in 4X/3X) < 0.05 and a corresponding logFC < 0 were depicted in green. Otherwise, they were designated in black.

### MicroRNA target prediction

Targets of miRNAs were predicted using psRNATarget with an expected value of 3 or less^98,107^. miRNAs that function through translation repression were filtered out while those that function through target cleavage were retained. Degradome sequencing data collected from various studies were used as a complement to show miRNA-target interactions^108–116^. Afterward, the gene model names of these targets were translated from B73 (AGPv4) to W22 (W22v2) using the Translate Gene Model IDs tool from MaizeGDB^117^. PCCs and the corresponding *P* values between expression levels of expressed miRNAs and their corresponding targets across the corresponding genotypes were computed (two-sided; confidence intervals, 95%; no adjustment made for multiple comparisons).

### GO term enrichment analysis

GO term enrichment analysis was performed using PANTHER online tools 17.0^118^. Genes of interest were translated from W22 (W22v2) to B73 (AGPv4) using the Translate Gene Model IDs tool from MaizeGDB^117^. Subsequently, genes were tested for over-or under-representation using Fisher’s exact test against all maize genes (AGPv4). Only significant terms (Bonferroni-corrected *P* values < 0.05) were listed (Supplementary Tables 3 and 4).

### Reporting summary

Further information on research design is available in the Nature Research Reporting Summary linked to this article.

## Supplementary information

Supplementary Information

Description of Additional Supplementary Files

Supplementary Data 1

Supplementary Data 2

Supplementary Data 3

Supplementary Data 4

Supplementary Data 5

Supplementary Data 6

Supplementary Data 7

Supplementary Data 8

Reporting Summary

## Data Availability

The sRNA-sequencing data generated in this study have been deposited in the Gene Expression Omnibus (GEO) repository under the accession code GSE189317. The processed data generated in this study are provided in the Supplementary Information file. The RefSeq database is available in NCBI [https://www.ncbi.nlm.nih.gov/refseq/] and the known miRNA sequences are available in the miRBase database [https://www.mirbase.org/]. The mRNA-sequencing data that support the findings of this study are available in the GEO repository under the accession code GSE149186. Source data are provided with this paper.

## Source data

Source Data
