# Supplementary material for: Dosage-sensitive miRNAs trigger modulation of gene expression during genomic imbalance in maize
Source: Nat Commun. 2022 May 31;13:3014. doi: 10.1038/s41467-022-30704-x (PMC9156689; doi:10.1038/s41467-022-30704-x)
Supplement: Supplementary file 1 — Supplementary Information [file 41467_2022_30704_MOESM1_ESM.pdf]

Dosage-sensitive miRNAs trigger modulation of gene expression during genomic imbalance in maize

Shi *et al.*

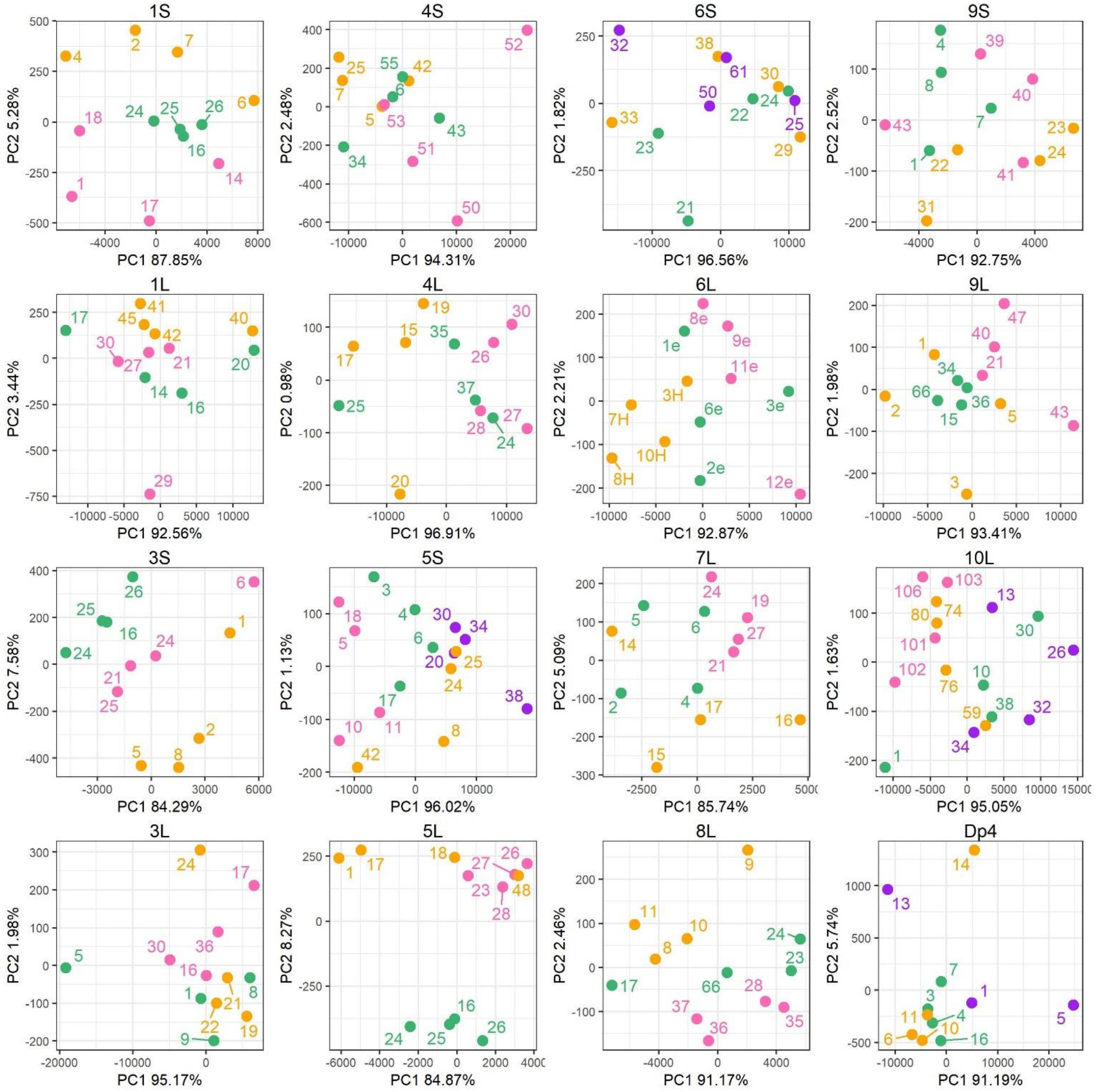

Supplementary Fig. 1. PCA plots of diploid aneuploids, including monosomies, trisomies, tetrasomies, and the corresponding diploid control. PCA plots were generated with normalized read counts. Data points of biological replicates from the same experimental or control group were labeled with their sample IDs (Supplementary Data 1). Each panel represents biological replicates from the experimental and control groups. Each group is labeled with a different color (1D, pink; 2D, green; 3D, orange; 4D, purple).

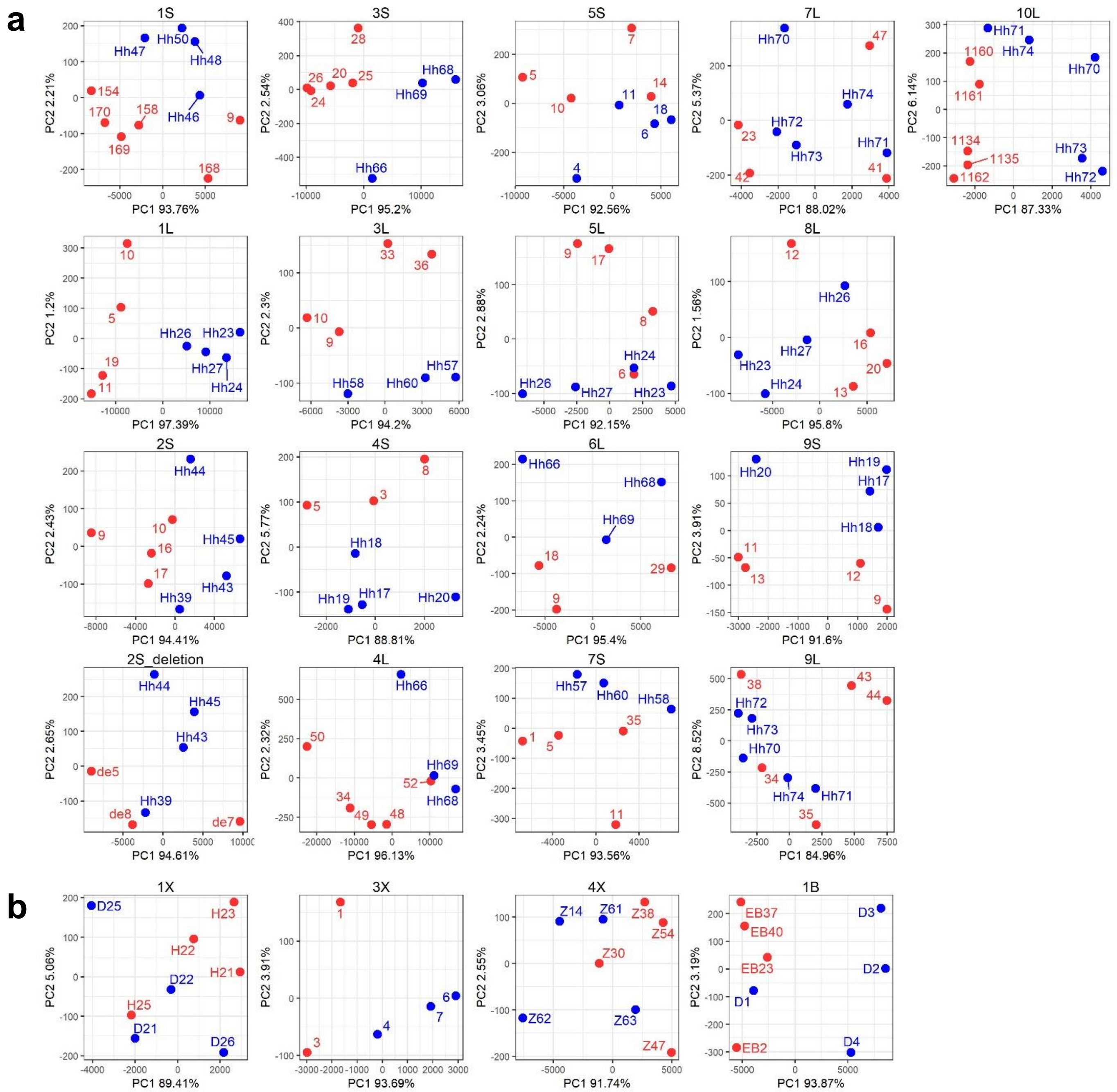

Supplementary Fig. 2. PCA plots of haploid aneuploids, polyploids, and diploids with one B chromosome. PCA plots were generated as described in Supplementary Fig. 1. **a** PCA plots of disomies (red) and the corresponding haploid control (blue). **b** PCA plots of polyploids and diploids with one B chromosome compared to diploids. The control group is labeled in blue (diploids) while the experimental group is labeled in red.

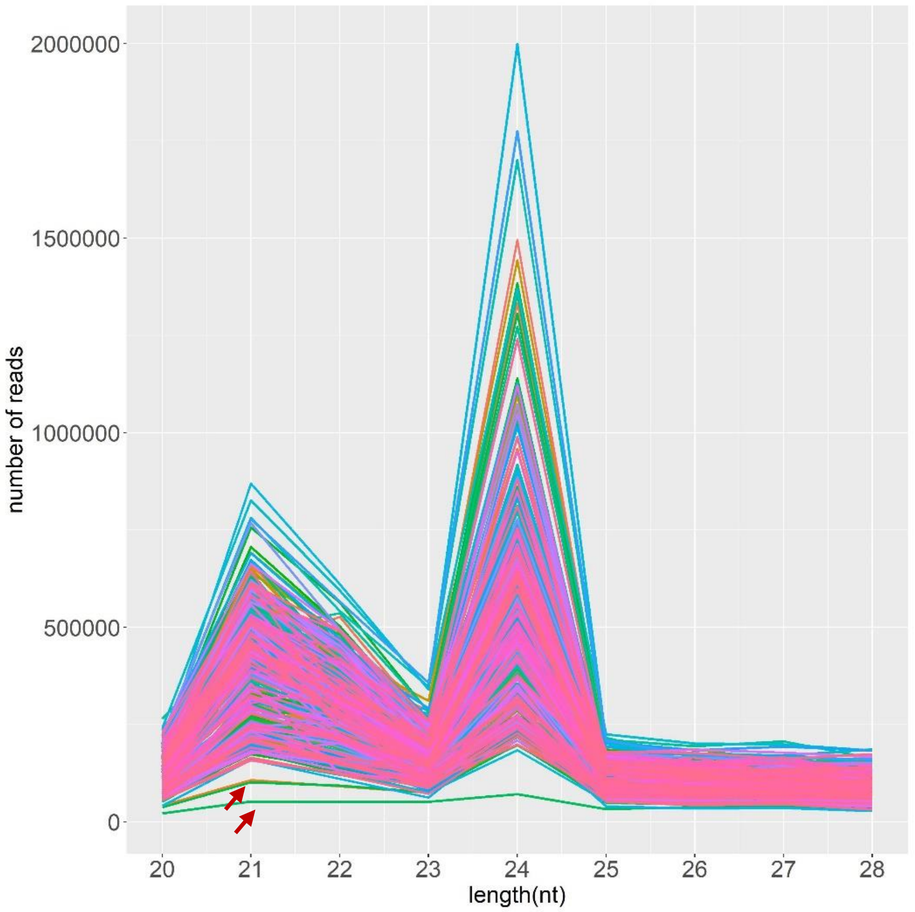

Supplementary Fig. 3. Read length distribution of sRNA libraries. Read length distribution of sRNA libraries after structural RNAs had been removed. Libraries that did not show distinct peaks at 21 and 24 nt are pointed by red arrows and were thus excluded from further analysis.

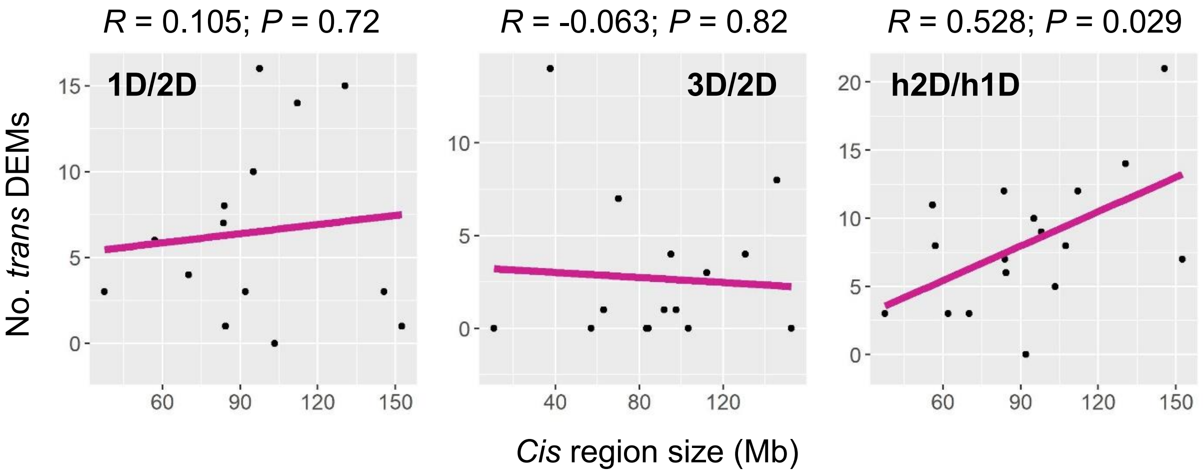

Supplementary Fig. 4. Correlation between the number of *trans* DEMs and the corresponding size of the *cis* region. The size of the *cis* region was computed in previous studies [1,2]. The number of DEMs was calculated as in Supplementary Data 3. *P*-value (*P*) represents the significance of Pearson correlation (two-sided; confidence intervals, 95%; no adjustment made for multiple comparisons), whereas *R* denotes the Pearson correlation coefficient.

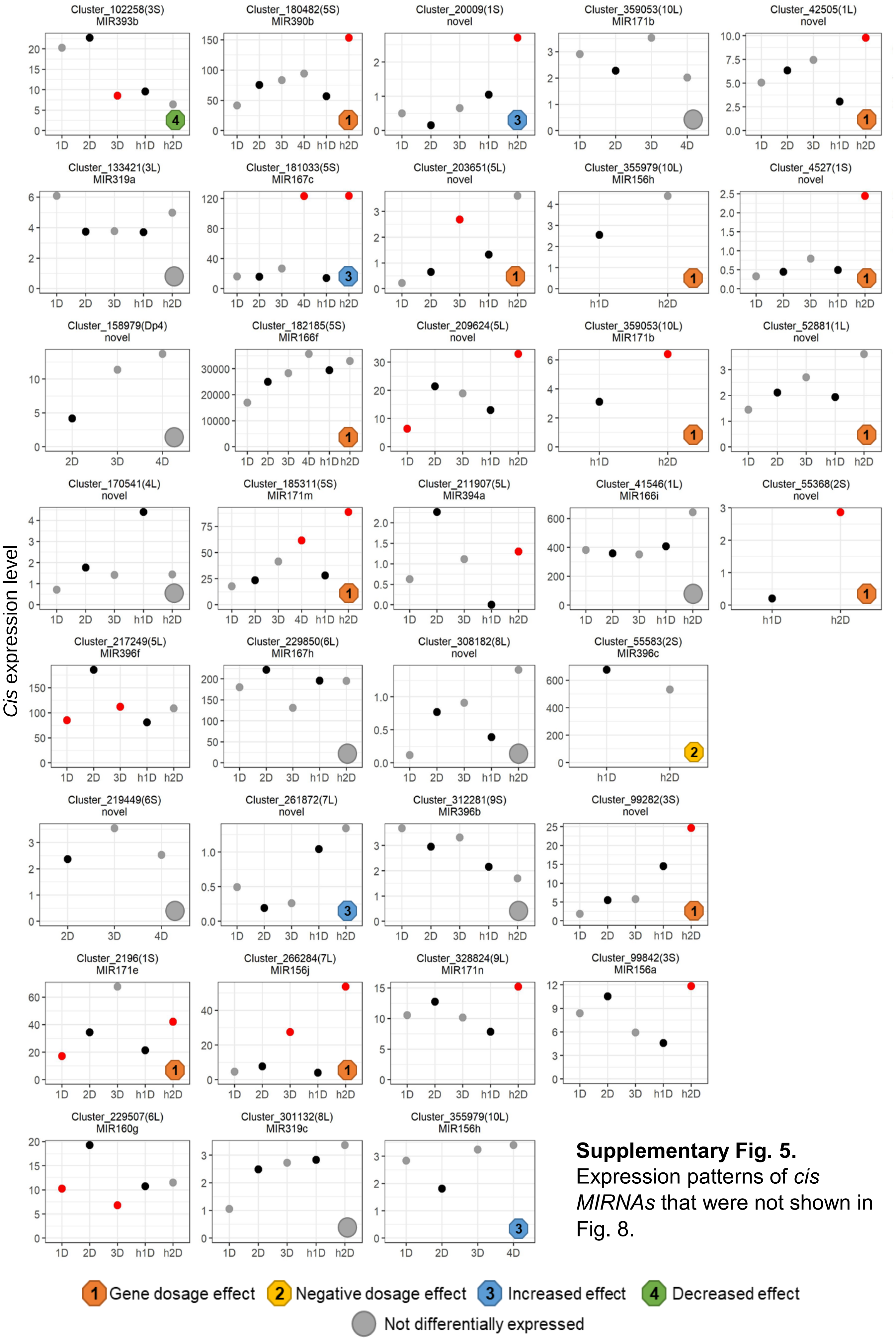

Supplementary Fig. 5. Expression patterns of *cis MIRNAs* that were not shown in Fig. 8.

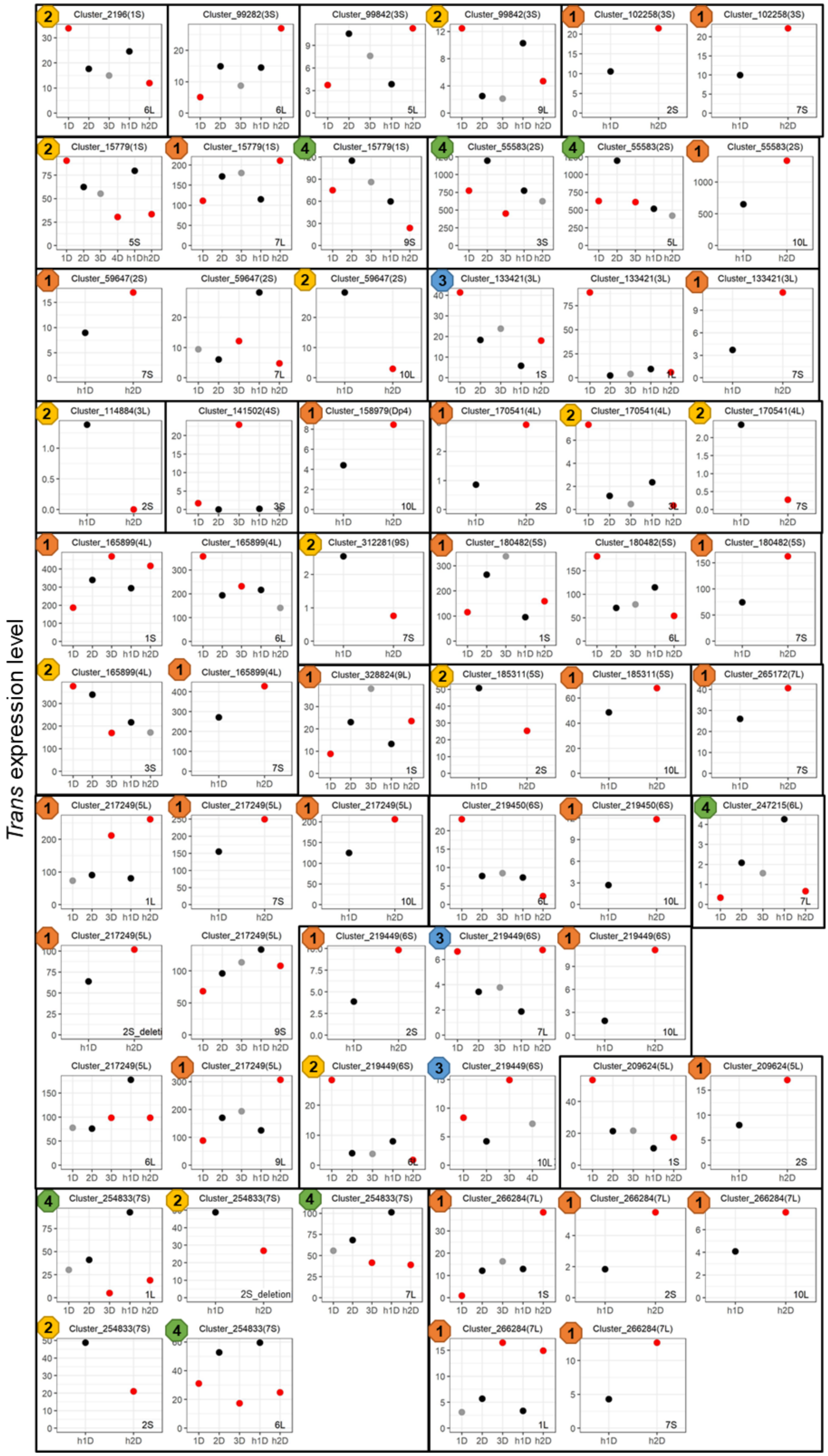

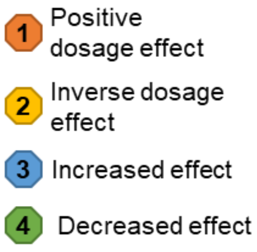

Supplementary Fig. 6. Expression patterns of *trans* DEMs. Plots were generated as in Fig. 9. The same DEMs expressed in *trans* with different *cis* regions are listed within the black box.

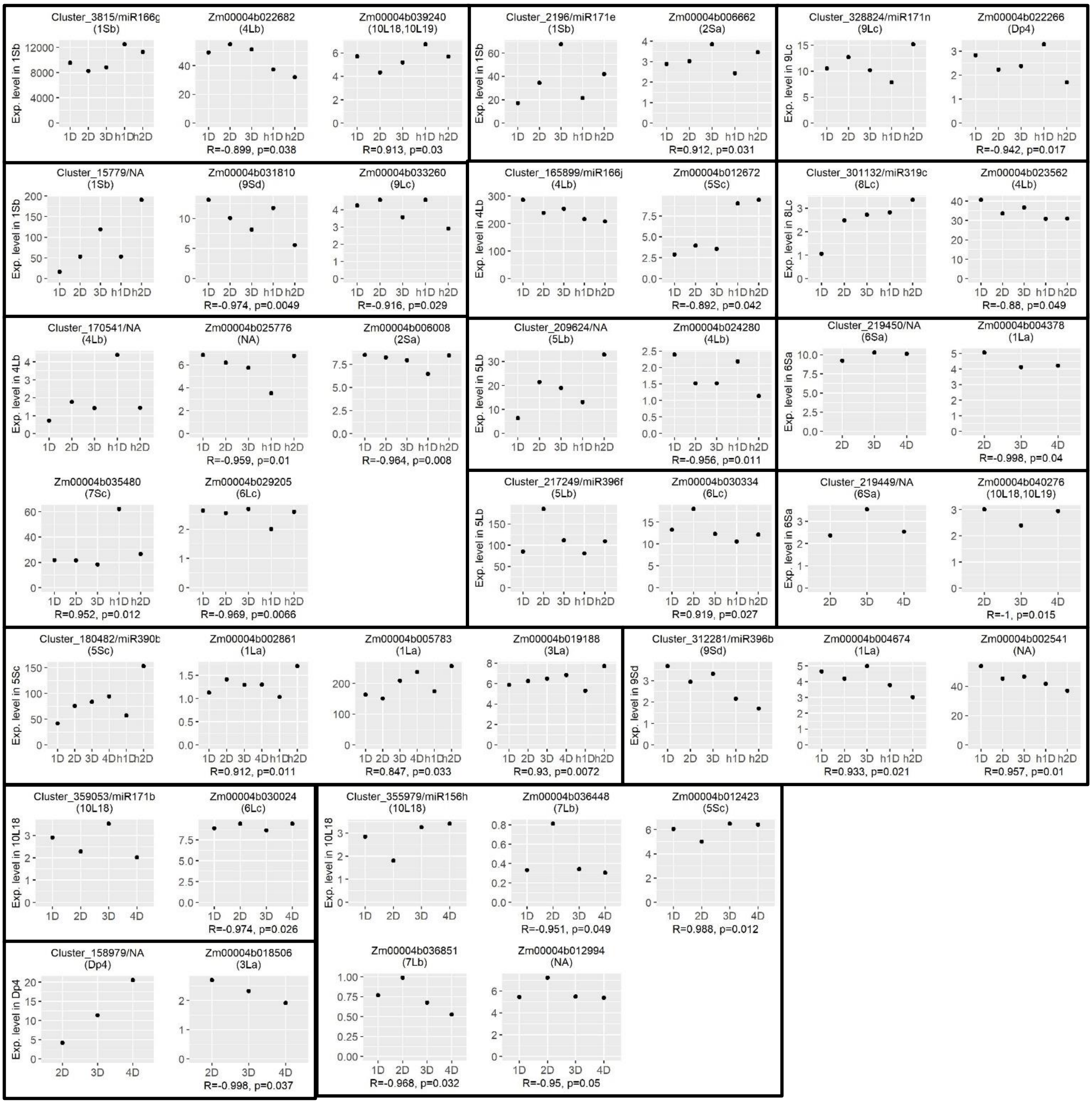

Supplementary Fig. 7. Expression patterns of *cis* miRNAs and their *trans* targets predicted by psRNATarget. The x-axis refers to the dosage of the chromosomal region as in the y-axis label, whereas the y-axis designates the expression level (rpm) of each *MIRNA* in *cis* or each predicted target in *trans* (normalized read counts) corresponding to the genotype on the x-axis. The *cis* location of each *MIRNA* or each target is depicted within parentheses. *R*, Pearson correlation coefficient between expression levels of each *MIRNA* and its corresponding target. p, *P*-values for Pearson correlation (two-sided; confidence intervals, 95%; no adjustment made for multiple comparisons).

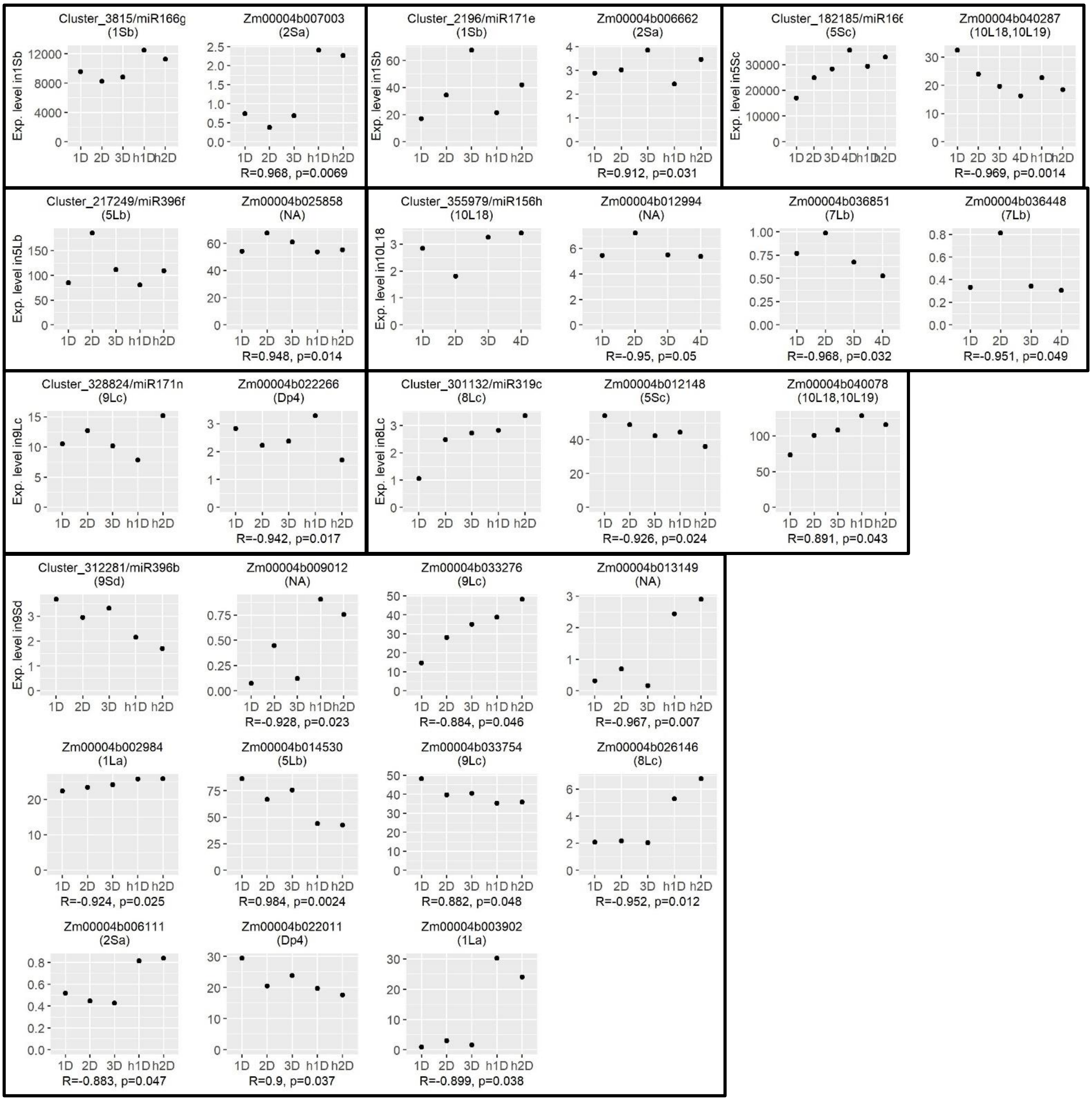

Supplementary Fig. 8. Expression patterns of *cis* miRNAs and their *trans* targets predicted by degradome sequencing plotted as in Supplementary Fig. 7.

Supplementary Table 1. The proportion of *trans* DEGs out of expressed *trans* genes (% *trans* DEGs) and that of *trans* DEMs out of expressed *trans* miRNAs (% *trans* DEMs) in each comparison. Data for differential gene expression were from previous studies [1,2]. Numbers in bold and underlined refer to the greater proportion out of % *trans* DEMs and % *trans* DEGs.

	1D/2D		3D/2D		4D/2D		h2D/h1D	
cis region	% *trans* DEG	% *trans* DEM	% *trans* DEG	% *trans* DEM	% *trans* DEG	% *trans* DEM	% *trans* DEG	% *trans* DEM
10L18	24.4%	**<u>37.2%</u>**	**<u>19.7%</u>**	2.3%	**<u>11.7%</u>**	7.0%		
10L19							**<u>47.8%</u>**	19.1%
1La	**<u>18.9%</u>**	6.8%	**<u>26.1%</u>**	13.6%			26.4%	**<u>47.7%</u>**
1Sb	37.4%	**<u>40.5%</u>**	**<u>17.2%</u>**	10.8%			11.8%	**<u>36.8%</u>**
2Sa_deletion							**<u>17.7%</u>**	7.0%
2Sa							**<u>26.2%</u>**	19.0%
3La	**<u>11.1%</u>**	2.4%	**<u>6.2%</u>**	0.0%			**<u>25.2%</u>**	16.3%
3Sb	**<u>39.5%</u>**	7.5%	**<u>36.3%</u>**	35.0%			**<u>20.3%</u>**	6.7%
4Lb	**<u>9.9%</u>**	2.4%	**<u>7.1%</u>**	0.0%			**<u>33.3%</u>**	13.0%
4Sa	**<u>12.9%</u>**	0.0%	**<u>4.0%</u>**	0.0%			**<u>12.7%</u>**	10.6%
5Lb	**<u>41.1%</u>**	11.1%	**<u>43.2%</u>**	19.4%			**<u>21.6%</u>**	7.3%
5Sc	5.8%	**<u>20.0%</u>**	**<u>2.2%</u>**	0.0%	9.3%	**<u>15.0%</u>**	**<u>24.8%</u>**	17.9%
6Lc	12.1%	**<u>35.0%</u>**	**<u>13.3%</u>**	7.5%			**<u>28.4%</u>**	25.0%
6Sa			**<u>1.1%</u>**	0.0%	**<u>2.3%</u>**	0.0%		
7Lb	4.0%	**<u>23.3%</u>**	4.1%	**<u>9.3%</u>**			**<u>33.8%</u>**	22.7%
7Sc							10.7%	**<u>25.0%</u>**
8Lc	1.3%	**<u>6.8%</u>**	2.0%	**<u>2.3%</u>**			**<u>11.0%</u>**	0.0%
9Lc	5.8%	**<u>15.6%</u>**	**<u>4.1%</u>**	0.0%			**<u>40.1%</u>**	25.0%
9Sd	**<u>9.6%</u>**	13.3%	**<u>5.7%</u>**	0.0%			11.3%	**<u>17.4%</u>**
Dp4			2.0%	**<u>2.3%</u>**	**<u>5.1%</u>**	0.0%		

Supplementary Table 2. Normalized read counts and *P*-values from DME analysis of all expressed *cis* miRNAs. DME analysis was performed by edgeR. Y, FDR < 0.05; N, FDR ≥ 0.05. Grey shades refer to data that is not available.

Cluster name	*Cis* region	Normalized read counts (rpm)						Statistically significant (Y/N)			
		1D	2D	3D	4D	h1D	h2D	1D/2D	3D/2D	4D/2D	h2D/h1D
Cluster_41546	1La	382.50	359.52	352.40		408.34	644.09	N	N		N
Cluster_42505	1La	5.07	6.33	7.44		3.07	9.79	N	N		Y
Cluster_52881	1La	1.45	2.11	2.70		1.94	3.61	N	N		N
Cluster_10766	1Sb	2.48	6.42	16.77		2.22	4.79	Y	Y		Y
Cluster_15779	1Sb	16.19	53.18	119.32		52.83	191.00	Y	Y		Y
Cluster_20009	1Sb	0.51	0.15	0.66		1.05	2.71	N	N		Y
Cluster_2196	1Sb	17.14	34.48	67.62		21.39	41.99	Y	N		Y
Cluster_3815	1Sb	9578.26	8241.52	8827.19		12524.63	11276.97	N	N		N
Cluster_4527	1Sb	0.33	0.45	0.80		0.50	2.44	N	N		Y
Cluster_55368	2Sa					0.20	2.87				Y
Cluster_55583	2Sa					677.16	533.60				N
Cluster_59647	2Sa					14.72	5.47				Y
Cluster_133421	3La	6.09	3.73	3.77		3.70	4.99	N	N		N
Cluster_102258	3Sb	20.28	22.74	8.56		9.59	6.42	N	Y		N
Cluster_99282	3Sb	1.89	5.49	5.74		14.51	24.68	N	N		Y
Cluster_99842	3Sb	8.37	10.55	5.92		4.59	11.85	N	N		Y
Cluster_165899	4Lb	286.58	238.70	253.90		216.52	207.89	N	N		N
Cluster_170541	4Lb	0.72	1.76	1.42		4.40	1.44	N	N		N
Cluster_203651	5Lb	0.22	0.65	2.68		1.32	3.61	N	Y		N
Cluster_209624	5Lb	6.41	21.42	18.90		13.03	32.93	Y	N		Y
Cluster_211907	5Lb	0.62	2.26	1.12		0.00	1.30	N	N		Y
Cluster_217249	5Lb	85.13	185.72	111.84		80.90	108.90	Y	Y		N
Cluster_180482	5Sc	41.61	75.47	83.43	94.13	56.92	153.36	N	N	N	Y
Cluster_181033	5Sc	16.24	15.79	26.93	123.26	14.04	123.65	N	N	Y	Y
Cluster_182185	5Sc	16990.12	24977.63	28304.42	35703.11	29384.64	32944.01	N	N	N	N
Cluster_185311	5Sc	17.64	23.62	41.54	61.64	28.15	88.88	N	N	Y	Y
Cluster_229507	6Lc	10.28	19.30	6.83		10.77	11.49	Y	Y		N
Cluster_229850	6Lc	179.99	221.78	131.21		195.61	195.55	N	N		N
Cluster_219449	6Sa		2.36	3.55	2.53				N	N	
Cluster_219450	6Sa		9.21	10.31	10.11				N	N	
Cluster_261872	7Lb	0.50	0.19	0.26		1.04	1.34	N	N		N
Cluster_265172	7Lb	19.31	20.80	26.48		21.05	24.93	N	N		N
Cluster_266284	7Lb	4.60	7.72	27.55		4.08	53.74	N	Y		Y
Cluster_273394	7Lb	1.43	4.88	9.62		5.16	17.41	Y	Y		Y
Cluster_254833	7Sc					93.45	42.21				Y
Cluster_301132	8Lc	1.06	2.49	2.73		2.83	3.37	N	N		N
Cluster_308182	8Lc	0.12	0.77	0.91		0.39	1.41	N	N		N
Cluster_328824	9Lc	10.53	12.73	10.16		7.84	15.22	N	N		Y
Cluster_312281	9Sd	3.69	2.95	3.32		2.16	1.70	N	N		N
Cluster_355979	10L18	2.84	1.81	3.25	3.41			N	N	N	
Cluster_359053	10L18	2.91	2.28	3.53	2.02			N	N	N	
Cluster_355979	10L19					2.55	4.40				N
Cluster_359053	10L19					3.11	6.39				Y
Cluster_158979	Dp4		4.19	11.36	20.58				N	N	
Cluster_161059	Dp4		2.06	1.94	1.94				N	N	

Supplementary Table 3. GO Enrichment analysis on *trans* mRNA targets of *cis* miRNAs, identified in psRNATarget prediction and/or degradome sequencing data analysis. Expression levels of these mRNA targets are significantly correlated with those of miRNAs.

**GO biological process complete**	Zea mays - REFLIST (39385)	input (233)	input (expected)	input (over/ under)	input (fold Enrichment)	input (FDR)
**protein refolding (GO:0042026)**	58	6	0.34	+	17.49	0.0048
**chaperone cofactor-dependent protein refolding (GO:0051085)**	71	6	0.42	+	14.29	0.0142
'de novo' posttranslational protein folding (GO:0051084)	71	6	0.42	+	14.29	0.0142
'de novo' protein folding (GO:0006458)	81	6	0.48	+	12.52	0.0289
**developmental process (GO:0032502)**	882	18	5.22	+	3.45	0.0164
regulation of RNA biosynthetic process (GO:2001141)	2761	44	16.33	+	2.69	4.69E-06
**regulation of transcription, DNA-templated (GO:0006355)**	2761	44	16.33	+	2.69	4.69E-06
regulation of nucleic acid-templated transcription (GO:1903506)	2761	44	16.33	+	2.69	4.69E-06
regulation of nucleobase-containing compound metabolic process (GO:0019219)	2981	45	17.63	+	2.55	1.53E-05
regulation of RNA metabolic process (GO:0051252)	2923	44	17.29	+	2.54	2.52E-05
regulation of cellular macromolecule biosynthetic process (GO:2000112)	3037	45	17.96	+	2.5	3.98E-05
regulation of macromolecule biosynthetic process (GO:0010556)	3043	45	18	+	2.5	4.09E-05
regulation of cellular biosynthetic process (GO:0031326)	3071	45	18.17	+	2.48	4.75E-05
regulation of biosynthetic process (GO:0009889)	3078	45	18.21	+	2.47	4.96E-05
regulation of gene expression (GO:0010468)	3383	46	20.01	+	2.3	0.000256
regulation of nitrogen compound metabolic process (GO:0051171)	3499	47	20.7	+	2.27	0.00024
regulation of primary metabolic process (GO:0080090)	3541	47	20.95	+	2.24	0.000467
regulation of cellular metabolic process (GO:0031323)	3589	46	21.23	+	2.17	0.0013
regulation of macromolecule metabolic process (GO:0060255)	3854	49	22.8	+	2.15	0.000787
regulation of metabolic process (GO:0019222)	3959	49	23.42	+	2.09	0.00204
regulation of cellular process (GO:0050794)	5014	57	29.66	+	1.92	0.00225
regulation of biological process (GO:0050789)	5556	63	32.87	+	1.92	0.000531
biological regulation (GO:0065007)	6277	69	37.13	+	1.86	0.000453
biological_process (GO:0008150)	22488	168	133.02	+	1.26	0.0063
Unclassified (UNCLASSIFIED)	16901	65	99.98	-	0.65	0

**Supplementary Table 4. GO Enrichment analysis on *trans* mRNA targets of *cis* miRNAs, identified in both psRNATarget prediction and degradome sequencing data analysis.** Expression levels of these mRNA targets are significantly correlated with those of miRNAs.						
**GO biological process complete**	Zea mays - REFLIST (39385)	input (33)	input (expected)	input (over/ under)	input (fold Enrichment)	input (FDR)
**transcription, DNA-templated (GO:0006351)**	400	10	0.34	+	29.84	2.18E-09
nucleic acid-templated transcription (GO:0097659)	411	10	0.34	+	29.04	2.83E-09
RNA biosynthetic process (GO:0032774)	423	10	0.35	+	28.22	3.74E-09
**developmental process (GO:0032502)**	882	10	0.74	+	13.53	4.24E-06
nucleobase-containing compound biosynthetic process (GO:0034654)	917	10	0.77	+	13.02	6.13E-06
heterocycle biosynthetic process (GO:0018130)	1157	10	0.97	+	10.32	5.44E-05
aromatic compound biosynthetic process (GO:0019438)	1170	10	0.98	+	10.2	6.03E-05
organic cyclic compound biosynthetic process (GO:1901362)	1320	10	1.11	+	9.04	0.000185
regulation of RNA biosynthetic process (GO:2001141)	2761	18	2.31	+	7.78	1.43E-09
**regulation of transcription, DNA-templated (GO:0006355)**	2761	18	2.31	+	7.78	1.43E-09
regulation of nucleic acid-templated transcription (GO:1903506)	2761	18	2.31	+	7.78	1.43E-09
regulation of RNA metabolic process (GO:0051252)	2923	18	2.45	+	7.35	3.73E-09
regulation of nucleobase-containing compound metabolic process (GO:0019219)	2981	18	2.5	+	7.21	5.19E-09
regulation of cellular macromolecule biosynthetic process (GO:2000112)	3037	18	2.54	+	7.07	7.09E-09
regulation of macromolecule biosynthetic process (GO:0010556)	3043	18	2.55	+	7.06	7.33E-09
RNA metabolic process (GO:0016070)	1861	11	1.56	+	7.06	0.000433
regulation of cellular biosynthetic process (GO:0031326)	3071	18	2.57	+	7	8.55E-09
regulation of biosynthetic process (GO:0009889)	3078	18	2.58	+	6.98	8.88E-09
regulation of nitrogen compound metabolic process (GO:0051171)	3499	19	2.93	+	6.48	5.77E-09
regulation of primary metabolic process (GO:0080090)	3541	19	2.97	+	6.4	7.12E-09
regulation of gene expression (GO:0010468)	3383	18	2.83	+	6.35	4.3E-08
regulation of cellular metabolic process (GO:0031323)	3589	19	3.01	+	6.32	9.03E-09
regulation of macromolecule metabolic process (GO:0060255)	3854	19	3.23	+	5.88	3.16E-08
regulation of metabolic process (GO:0019222)	3959	19	3.32	+	5.73	5.05E-08
cellular nitrogen compound biosynthetic process (GO:0044271)	2091	10	1.75	+	5.71	0.0119
cellular macromolecule biosynthetic process (GO:0034645)	2159	10	1.81	+	5.53	0.0157
macromolecule biosynthetic process (GO:0009059)	2223	10	1.86	+	5.37	0.0203
gene expression (GO:0010467)	2475	11	2.07	+	5.3	0.00712
regulation of cellular process (GO:0050794)	5014	20	4.2	+	4.76	3.03E-07
nucleic acid metabolic process (GO:0090304)	2894	11	2.42	+	4.54	0.0316
regulation of biological process (GO:0050789)	5556	20	4.65	+	4.3	1.93E-06
biological regulation (GO:0065007)	6277	21	5.26	+	3.99	1.97E-06
Unclassified (UNCLASSIFIED)	16901	9	14.16	-	0.64	0

Supplementary references

1. Shi, X. *et al.* Genomic imbalance determines positive and negative modulation of gene expression in diploid maize. *Plant Cell* **33**, 917–939 (2021).
2. Yang, H. *et al.* Predominantly inverse modulation of gene expression in genomically unbalanced disomic haploid maize. *Plant Cell* **33**, 901–916 (2021).
