# Supplementary material for: Dosage-sensitive miRNAs trigger modulation of gene expression during genomic imbalance in maize
Source: Nat Commun. 2022 May 31;13:3014. doi: 10.1038/s41467-022-30704-x (PMC9156689; doi:10.1038/s41467-022-30704-x)
Supplement: Supplementary file 2 — Description of Additional Supplementary Files [file 41467_2022_30704_MOESM2_ESM.pdf]

Description of Additional Supplementary Files

File Name: Supplementary Data 1
Description: Read mapping statistics and grouping information of each sRNA-seq experiment.

File Name: Supplementary Data 2
Description: Information of MIRNA loci identified by ShortStack.

File Name: Supplementary Data 3
Description: The number of DEMs in each comparison.

File Name: Supplementary Data 4
Description: Mean expression levels of identified MIRNA loci and DME analysis for each MIRNA loci.

File Name: Supplementary Data 5
Description: Mean, median, SD for ratios of miRNAs with extreme values (ratio >6 or < 1/6) excluded.

File Name: Supplementary Data 6
Description: Predicted targets of miRNAs identified by psRNATarget.

File Name: Supplementary Data 7
Description: Significant correlations between expression levels of miRNAs and their mRNA targets predicted by psRNATarget.

File Name: Supplementary Data 8
Description: Significant correlations between expression levels of miRNAs and their mRNA targets found in degradome sequencing data.
