# Supplementary material for: Dosage-sensitive miRNAs trigger modulation of gene expression during genomic imbalance in maize
Source: Nat Commun. 2022 May 31;13:3014. doi: 10.1038/s41467-022-30704-x (PMC9156689; doi:10.1038/s41467-022-30704-x)
Supplement: Supplementary file 11 — Reporting Summary [file 41467_2022_30704_MOESM11_ESM.pdf]

nature portfolio

Corresponding author(s): James A. Birchler

Last updated by author(s): Apr 28, 2022

Reporting Summary

Nature Portfolio wishes to improve the reproducibility of the work that we publish. This form provides structure for consistency and transparency in reporting. For further information on Nature Portfolio policies, see our Editorial Policies and the Editorial Policy Checklist.

Statistics

For all statistical analyses, confirm that the following items are present in the figure legend, table legend, main text, or Methods section.

n/a	Confirmed	
☐	☒	The exact sample size (*n*) for each experimental group/condition, given as a discrete number and unit of measurement
☐	☒	A statement on whether measurements were taken from distinct samples or whether the same sample was measured repeatedly
☐	☒	The statistical test(s) used AND whether they are one- or two-sided<br>*Only common tests should be described solely by name; describe more complex techniques in the Methods section.*
☒	☐	A description of all covariates tested
☐	☒	A description of any assumptions or corrections, such as tests of normality and adjustment for multiple comparisons
☐	☒	A full description of the statistical parameters including central tendency (e.g. means) or other basic estimates (e.g. regression coefficient) AND variation (e.g. standard deviation) or associated estimates of uncertainty (e.g. confidence intervals)
☐	☒	For null hypothesis testing, the test statistic (e.g. *F*, *t*, *r*) with confidence intervals, effect sizes, degrees of freedom and *P* value noted<br>*Give P values as exact values whenever suitable.*
☒	☐	For Bayesian analysis, information on the choice of priors and Markov chain Monte Carlo settings
☒	☐	For hierarchical and complex designs, identification of the appropriate level for tests and full reporting of outcomes
☐	☒	Estimates of effect sizes (e.g. Cohen's *d*, Pearson's *r*), indicating how they were calculated

Our web collection on statistics for biologists contains articles on many of the points above.

Software and code

Policy information about availability of computer code

Data collection	No software was used.
Data analysis	cutadapt version 1.16, FASTX-Toolkit version 0.0.13, bowtie2-2.2.9, ShortStack version 3.8.3, BLAST version 2.12.0, 'edgeR' version 3.30.3, psRNATarget (2017), PANTHER online tool version 17.0

For manuscripts utilizing custom algorithms or software that are central to the research but not yet described in published literature, software must be made available to editors and reviewers. We strongly encourage code deposition in a community repository (e.g. GitHub). See the Nature Portfolio guidelines for submitting code & software for further information.

Data

Policy information about availability of data

All manuscripts must include a data availability statement. This statement should provide the following information, where applicable:

- Accession codes, unique identifiers, or web links for publicly available datasets
- A description of any restrictions on data availability
- For clinical datasets or third party data, please ensure that the statement adheres to our policy

The sRNA-sequencing data generated in this study have been deposited in the Gene Expression Omnibus (GEO) repository under the accession code GSE189317 [https://www.ncbi.nlm.nih.gov/geo/query/acc.cgi?&acc=GSE189317]. The processed data generated in this study are provided in the Supplementary Information/ Source Data file. The RefSeq database is available in NCBI [https://www.ncbi.nlm.nih.gov/refseq/] and the known miRNA sequences are available in the miRBase database [https://www.mirbase.org/]. The mRNA-sequencing data that support the findings of this study are available in the GEO repository under the accession code GSE149186 [https://www.ncbi.nlm.nih.gov/geo/query/acc.cgi?acc=GSE149186].

Field-specific reporting

Please select the one below that is the best fit for your research. If you are not sure, read the appropriate sections before making your selection.

- [x] Life sciences
- [ ] Behavioural & social sciences
- [ ] Ecological, evolutionary & environmental sciences

For a reference copy of the document with all sections, see nature.com/documents/nr-reporting-summary-flat.pdf

Life sciences study design

All studies must disclose on these points even when the disclosure is negative.

Sample size	Four biological replicates were intended to be used for each genotype, which provides a balance between statistical soundness and reasonable costs. However, due to the limitation of materials, for a few genotypes, 2-3 biological replicates were used. PCA analysis shows that none of the biological replicate should be considered as an outlier. In addition, normalized expression levels of each pair of biological replicates are significantly correlated. Therefore, a low level of variability between biological replicates was detected. Furthermore, differential gene expression analysis has detected a reasonable number of significantly differentially expressed miRNAs. All these observations support that 4 biological replicates are sufficient.
Data exclusions	The read length distribution of three sRNA libraries after structural RNAs had been removed did not show distinct peaks at 21 and 24 nt, indicating the lower quality of data. Thus, these samples (4Dp22C5, 1La20, and 1La40) were excluded from further study. In addition, for calculating the Pearson correlation coefficient in Figure 1, data points with x- and y-axis values exceeding two SDs from the mean are considered as outliers, thus are excluded in the analysis.
Replication	The data processing was done twice, and the replication was successful.
Randomization	Samples were allocated into experimental groups according to their genotypes. All plants were grown in the green house and their tissue was collected 45 days after germination at the same time of the day to control for covariates. Randomization is not relevant in this study because the major difference between the treatment and the control lies in their genotypes.
Blinding	Blinding is not relevant in this experiment as there are no trials or treatments.

Reporting for specific materials, systems and methods

We require information from authors about some types of materials, experimental systems and methods used in many studies. Here, indicate whether each material, system or method listed is relevant to your study. If you are not sure if a list item applies to your research, read the appropriate section before selecting a response.

Materials & experimental systems

n/a	Involved in the study
[x]	[ ] Antibodies
[x]	[ ] Eukaryotic cell lines
[x]	[ ] Palaeontology and archaeology
[x]	[ ] Animals and other organisms
[x]	[ ] Human research participants
[x]	[ ] Clinical data
[x]	[ ] Dual use research of concern

Methods

n/a	Involved in the study
[x]	[ ] ChIP-seq
[x]	[ ] Flow cytometry
[x]	[ ] MRI-based neuroimaging
